# Lower mortality after early supervised pulmonary rehabilitation following COPD-exacerbations: a systematic review and meta-analysis

**DOI:** 10.1186/s12890-018-0718-1

**Published:** 2018-09-15

**Authors:** Camilla Koch Ryrsø, Nina Skavlan Godtfredsen, Linette Marie Kofod, Marie Lavesen, Line Mogensen, Randi Tobberup, Ingeborg Farver-Vestergaard, Henriette Edemann Callesen, Britta Tendal, Peter Lange, Ulrik Winning Iepsen

**Affiliations:** 1 The Centre of Inflammation and Metabolism and the Centre for Physical Activity Research, Rigshospitalet, University of Copenhagen, Blegdamsvej 9, DK-2100 Copenhagen, Denmark; 2 Danish Health Authority, Copenhagen, Denmark; 3 Department of Respiratory Medicine, Copenhagen University Hospital, Hvidovre, Denmark; 4 Department of Clinical Medicine, University of Copenhagen, Copenhagen, Denmark; 5 Department of Physiotherapy, Copenhagen University Hospital, Hvidovre, Denmark; 6 Department of Pulmonary and Infectious Diseases, Copenhagen University Hospital, Nordsjælland, Hillerød, Denmark; 7 The Department of the Elderly and Disabled, Odense Municipality, Odense, Denmark; 8 Department of Gastroenterology, Center for Nutrition and Bowel Disease, Aalborg University Hospital, Aalborg, Denmark; 9 Unit for Psychooncology and Health Psychology, Aarhus University Hospital and Aarhus University, Aarhus, Denmark; 10 Department of Public Health, Section of Social Medicine, University of Copenhagen, Copenhagen, Denmark; 11 Medical Department O, Respiratory Section, Herlev and Gentofte Hospital, Herlev, Denmark

**Keywords:** Chronic obstructive pulmonary disease, Supervised early pulmonary rehabilitation, Exacerbation of COPD, Hospital readmissions, Mortality, Systematic review

## Abstract

**Background:**

Pulmonary rehabilitation (PR), delivered as a supervised multidisciplinary program including exercise training, is one of the cornerstones in the chronic obstructive pulmonary disease (COPD) management. We performed a systematic review and meta-analysis to assess the effect on mortality of a supervised early PR program, initiated during or within 4 weeks after hospitalization with an acute exacerbation of COPD compared with usual post-exacerbation care or no PR program. Secondary outcomes were days in hospital, COPD related readmissions, health-related quality of life (HRQoL), exercise capacity (walking distance), activities of daily living (ADL), fall risk and drop-out rate.

**Methods:**

We identified randomized trials through a systematic search using MEDLINE, EMBASE and Cocharne Library and other sources through October 2017. Risk of bias was assessed regarding randomization, allocation sequence concealment, blinding, incomplete outcome data, selective outcome reporting, and other biases using the Cochrane Risk of Bias tool.

**Results:**

We included 13 randomized trials (801 participants). Our meta-analyses showed a clinically relevant reduction in mortality after early PR (4 trials, 319 patients; RR = 0.58 (95% CI: [0.35 to 0.98])) and at the longest follow-up (3 trials, 127 patients; RR = 0.55 (95% CI: [0.12 to 2.57])). Early PR reduced number of days in hospital by 4.27 days (1 trial, 180 patients; 95% CI: [− 6.85 to − 1.69]) and hospital readmissions (6 trials, 319 patients; RR = 0.47 (95% CI: [0.29 to 0.75])). Moreover, early PR improved HRQoL and walking distance, and did not affect drop-out rate. Several of the trials had unclear risk of bias in regard to the randomization and blinding, for some outcome there was also a lack of power.

**Conclusion:**

Moderate quality of evidence showed reductions in mortality, number of days in hospital and number of readmissions after early PR in patients hospitalized with a COPD exacerbation. Long-term effects on mortality were not statistically significant, but improvements in HRQoL and exercise capacity appeared to be maintained for at least 12 months. Therefore, we recommend early supervised PR to patients with COPD-related exacerbations. PR should be initiated during hospital admission or within 4 weeks after hospital discharge.

**Electronic supplementary material:**

The online version of this article (10.1186/s12890-018-0718-1) contains supplementary material, which is available to authorized users.

## Background

Acute exacerbation in chronic obstructive pulmonary disease (AECOPD) is the most common reason for hospital admission among patients with chronic obstructive pulmonary disease (COPD) [1]. These events result in higher mortality and lower quality of life [2]. The estimated mortality rate within 90 days after hospitalization for AECOPD is approximately 3.6% (1.8–20.4%) while mortality rate during the first 2 years after admission for AECOPD is approximately 31.0% (18.8–45.4%) [3]. The estimated 30-day and 12-month readmission-rate after AECOPD hospitalization is approximately 19.2% [4] and 42.3% [5], respectively. Readmission following an AECOPD has a negative effect on physical performance by lowering exercise capacity, muscle strength and physical activity level, which patients may never fully recover from [6, 7]. Patients with frequent exacerbations may be prone to a more rapid decline in activities of daily living (ADL) and functional capacity, which is associated with reductions health-related quality of life (HRQoL) [6]. Repeated exacerbations may cause a *vicious circle* as physical inactivity and low exercise capacity are related to a higher risk of hospital readmission, regardless of the COPD severity [8].

Pulmonary rehabilitation (PR) has been suggested in AECOPD because of its known beneficial effects on exercise capacity, HRQoL and symptom burden in stable patients [9, 10]. It should be noted that the evidence in favor of PR in stable COPD is based on studies investigating supervised PR programs including exercise training for 6–12 weeks [11, 12], although at long-term follow-up, adherence to exercise training is low and effects are not maintained [13]. Likewise, studies have shown that early PR, initiated at the beginning of exacerbation treatment or within 3 weeks of initiation of exacerbation treatment, improves exercise capacity and HRQoL along with reductions in hospital readmissions [14] and mortality [15] compared with no PR. Based on evidence from randomized controlled trials (RCT), NICE guidelines from 2011 recommended the use of early PR in patients hospitalized with COPD exacerbations [16]. Yet, recent concerns have been raised about PR not being safe in AECOPD when initiated during the hospital admission [17]. Based on this new evidence, the 2017 guideline from the European Respiratory Society (ERS) and American Thoracic Society (ATS) recommend that PR is not initiated during hospitalization in patients with COPD related exacerbations, but is delayed until after hospital discharge (within 3 weeks) [18]. However, the ERS/ATS recommendation was based on both supervised and unsupervised PR programs, and interestingly, the potentially negative effects of early PR were mainly driven by studies providing unsupervised PR.

Therefore, our aim was to investigate the effect of a supervised early PR program, initiated during or within 4 weeks, in patients hospitalized with a COPD exacerbation compared with usual care. Our primary outcome was mortality at the end of PR and at the longest follow-up. Secondary outcomes were hospital readmission, days in hospital, HRQoL and exercise capacity. We followed the guidelines of the Grading of Recommendations Assessment, Development and Evaluation (GRADE) Working Group [19] in order to support clinical decision making in a national Danish setting where only supervised PR programs take place.

## Methods

### Protocol and registration

This review was among a series of reviews performed for a guideline developed by the Danish Health Authority. The population, intervention, control intervention (comparison) as well as critical and important outcomes (PICO) [20] were determined by the working-group members prior to our literature search. The methods and review process are a standardized part of the guideline development process within the Danish Health Authority. The methods handbook (in Danish) as well as the full guideline and more detail regarding the PICO can be accessed at www.sst.dk, the full guideline can also be found on https://app.magicapp.org/app#/guideline/2551.

### Eligibility criteria

We considered studies eligible if they compared the effect of early supervised PR initiated during admission or within 4 weeks of hospital discharge (intervention) with no early pulmonary rehabilitation/usual care (comparison) in patients admitted and/or having been admitted to hospital with exacerbations of COPD (population). The PR was defined by a main component of supervised exercise training but could contain education, smoking cessation, nutritional support, management in activities of daily living (ADL) and physio-social support.

Studies providing inpatient pulmonary rehabilitation with exercise training was included if rehabilitation were continued after hospital discharge and/or a comprehensive rehabilitation program could be documented. Studies were excluded if they were not randomized or did not cover the predefined PICO. Our pre-specified outcomes were evaluated immediately after the end of intervention and at the longest follow up. Our primary outcome was mortality while secondary outcomes included number of days in hospital, number of COPD related hospital readmissions, health related quality of life (HRQoL), exercise capacity (walking tests), activities of daily living (ADL), falls and dropout.

### Information sources and search strategy

A research librarian and search specialist performed the systematic literature search including the following databases: Medline, Embase, Physiotherapy Evidence Database (PEDRO), CINAHL, G-I-N international, NICE, National Guideline Clearinghouse, Surgical Implant Generation Network, Cochrane Library and OTseeker. The full search strategy is presented in Additional file 1.

**Fig. 1:**
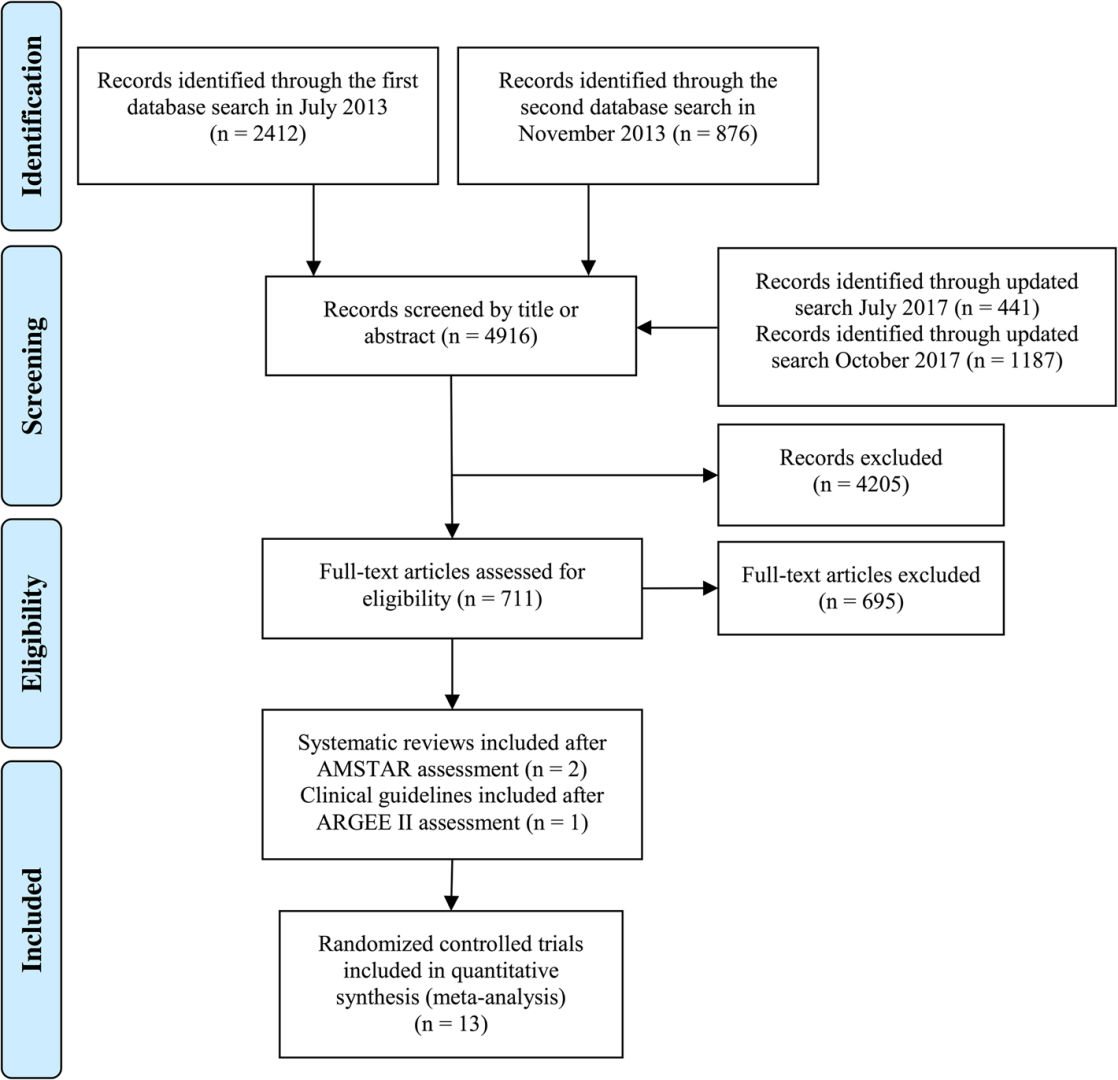
PRISMA (Preferred Reporting Items for Systematic Reviews and Meta-Analyses) flow diagram of the article selection processes

This review is an update of a previous review. First, a comprehensive search for COPD rehabilitation guidelines and systematic reviews was conducted in July 2013, yielding a total of 2412 records. In November 2013, a second and more specific search (Medline, Embase and PEDRO) for RCTs was performed, in which 876 additional records were identified. An updated search for guidelines and systematic reviews was conducted in July 2017, covering the period from July 2013 to July 2017, where we identified 460 additional records. The search for primary studies was updated in October 2017, covering the period from December 2013 to October 2017. The search resulted in 1187 additional records (Fig. 1). All records were screened for relevant titles or abstracts, while reference lists of included studies were assessed for further eligible literature.

### Study selection

Clinical guidelines identified in the first search were evaluated with the Appraisal of Guidelines for Research and Evaluation instrument version II (AGREE II) by two independent authors and disagreement was resolved through consensus (see Additional file 2). Likewise, systematic reviews were assessed with A Measurement Tool to Asses Systematic Reviews (AMSTAR) by three authors and disagreement was resolved through consensus (see Additional file 3). Based on these assessments we decided to include one clinical guideline [21] and two systematic reviews [14, 15]. From the second search, two authors independently evaluated the full text of all potentially eligible studies and decided whether to include or exclude each study based on the prespecified criteria.

### Data analysis and risk of bias

Data on participants, study design, interventions and outcomes were extracted from the full-text reports of the included studies by two independent authors, using Covidence (Covidence systematic review software, Veritas Health Innovation, Melbourne, Australia. Available at www.covidence.org). Disagreement was resolved through consensus. Each included study was assessed using the Cochrane risk of bias tool [22]. Two independent authors performed the risk of bias assessment, and disagreement was resolved through discussion and consensus (see Additional file 4).

We used mean difference (MD) to calculate effect estimates for continuous outcomes if the same scale was used for a particular outcome. When pooling continuous outcome data from different scales a standardized mean difference (SMD) was calculated. Rate ratio and relative risk (RR) was used to calculate effects for dichotomous outcomes. Random-effects meta-analyses were performed as we expected variation in population, duration of intervention, and types of training between the included studies. Review Manager 5.3 software [23] was used for the statistical analyses and to produce forest plots. Heterogeneity in the effect estimate was determined using the I-square (*I*^*2*^*)* statistic and values below 40% indicated low heterogeneity [24].

The quality of evidence for each outcome was assessed across the included studies as proposed by the GRADE Working Group [25]. A draft of the grading for each outcome using the GRADE criteria (i.e., risk of bias, inconsistency, indirectness, imprecision, and publication bias) was presented to the working group and the final grading was reached through discussion and consensus. The full guideline was then submitted to peer review and public hearing. For details on the hearing see www.sst.dk (in Danish).

### Assessment of PR extensiveness

We assessed the extensiveness of the PR program in the included trials by following the statements and guidelines from BTS [26], ERS/ATS [10]), and as described in Puhan et al. [14] (see Additional file 5).

## Results

### Study selection

We identified 13 eligible primary RCTs for our analysis. These included a total of 801 participants who were in the recovery phase of a recent COPD exacerbation. Excluding dropouts (167 participants), 634 participants were included in our analysis. Nine of the 13 studies were included in a systematic Cochrane review [14]. Figure 1 summarizes the flow diagram of the two selection processes.

### Included studies

Table 1 shows the characteristics of the included studies. In three studies [27–29] patients initiated an inpatient PR program within 4 to 8 days of hospital admission. In one study [30] patients began PR as either in- or outpatients and all continued as outpatients, in eight studies [31–38] the outpatient program was initiated within one to four weeks after the inpatient exacerbation treatment and in one study [39] the outpatient rehabilitation was initiated after the “hospital at home” treatment of the exacerbation. In four studies [27, 29, 38, 39] the PR consisted of only supervised exercise training, whereas in the remaining nine studies [28, 30–37] PR consisted of supervised exercise training and education, smoking cessation, nutritional support, management in activities of daily living (ADL) and physio-social support. Duration of the different PR programs was ten days to six months, with training frequencies ranging from two to seven times a week, and exercise durations of 30–90 min per session. Table S1 in the Additional file 5 shows the extensiveness of the PR programs in the included trials. The participants followed an extensive PR program in ten of the included trials [27–31, 33–36, 38]. In the remaining three studies, the extensiveness of the PR was deemed as moderate [39], slightly extensive [37], and undescribed [32]. The control group received usual care consisting of optimal medical treatment. There were no reported differences in baseline characteristics of patients between groups in all of the included studies.

**Table 1:** Characteristics of the included studies

Reference	Country	Study design	Setting, duration and frequency	Participants	Intervention	Intervention after discharge	Usual care	Notes	Outcomes	Dropouts
Behnke 2000 [27]	Germany	RCT	Setting: in- and outpatient Duration: hospital-based 10 days, home-based 6 months Frequency: 7/week	46 admitted patients with AECOPD (mean age: 64–68 years, FEV_1_: 36% predicted). Comorbidities: not specified.	PR consisted of conventional therapy including 30 min of daily breath exercises with respirologists and hospital-based training. Exercise training consisted of daily 6MWT and 5 self-controlled walking sessions at 75% of the treadmill walking distance of the respective day.	Supervised home-based training for 6 mo.: walking training 3/day at 125% of the best 6MWD, health check every 2 weeks (mo. 0–3) followed by phone calls from mo. 3–6.	Usual care: standard inpatient care and community care with respirologists (30 min of daily breathing exercises) but without exercise training	Both groups (intervention and usual care) were supervised by the physician.	Mortality^b^ Walking test^b^ COPD related hospital readmissions^b^ Dropout^a^	16 dropouts (8 in PR group and 8 in control group)
Daabis 2017 [31]	Egypt	RCT	Setting: outpatient Duration: 8 weeks Frequency: 3/week	30 admitted patients with AECOPD (mean age: 58–61 years, FEV_1_: 53–56% of predicted). Comorbidities: not specified.	PR consisted of patient assessment, exercise training (ET), patient education including self-management of the disease, nutrition and lifestyle issues. Exercise training consisted of ET with 30-min of walking at the intensity of 75% 6MWT including 30-min of low-intensity RT.	Outpatient PR	Medical treatment.	All patients received standard treatment with optimal medical treatment.	HRQoL^a^ Walking distance^a^	No dropouts reported
Deepak 2014 [32]	India	RCT	Setting outpatient Duration: 12 weeks	60 admitted patients with AECOPD (mean age: 59 years, FEV_1_: 47–53% of predicted, 93% men). Comorbidities: not specified.	PR consisted of patient assessment, exercise testing, exercise training (mixture of limb strengthening and aerobic activities, tailored to individual baseline function), education, nutrition and psycho-social rehabilitation.	Outpatient PR	Conventional treatment without PR.	All patients received conventional management consisting of medical treatment.	HRQoL^a^ Walking distance^a^	4 dropouts
Eaton 2009 [28]	New Zealand	RCT	Setting: in- and outpatient Duration: 8 weeks Frequency: 2/week	97 admitted patients with AECOPD (mean age: 70 years, FEV_1_: 35–36% of predicted, 42–45% men). Comorbidities: Measured with Charlson index (PR group: 3.1; control: 3.2).	PR consisted of a daily 30-min structured, supervised exercise regimen that included walking and upper and lower limb strengthening exercises.	Hospital-based outpatient program consisting of 1-h sessions of supervised exercise training and educational sessions (e.g. coping with dyspnea, management of ADL, nutritional advises, airway clearance).	Usual care standardized in according with the ATS/ERS COPD guidelines and standardized advises on the benefits of exercise and maintaining daily activities.	All patients received usual care standardized in according with the ATS/ERS COPD guidelines.	Walking distance^a^ COPD related hospital readmissions^b^ Dropout^a^	13 dropouts (8 in PR group and 5 in control group)
Kirsten 1998 [29]	Germany	RCT	Setting: inpatient Duration: 10 days Frequency: 7/week	31 admitted patients with AECOPD (mean age: 62–66 years, FEV_1_: 34–38% of predicted, 90% men). Comorbidities: not specified.	PR consisted of 6MWT each day and additional 5 walking sessions per day at ≥75% of the respective walking distance.	Inpatient supervised walking sessions 5/day.	Usual care with optimal medical treatment.	All patients received standard medical treatment.	Walking test^a^	2 dropouts (not reported in which group)
Ko 2011 [34]	China	RCT	Setting: outpatient Duration: 8 weeks Frequency: 3/week	60 admitted patients with AECOPD (mean age: 73–74 years, FEV_1_: 41–46% of predicted, 98% men). Comorbidities: coronary artery disease, cardiac arrhythmic, heart failure, hypertension, diabetes.	PR consisted of supervised exercise training including treadmill, arm cycling, arm and leg strength training at 60–70% of VO_2max_ or HR_max_ and were advised to perform at least 20 min home exercises a day. Education on proper breathing techniques and how to cope with daily activities.	Supervised outpatient exercise training.	Usual care with instructions to perform regular exercise at home (walking and muscle stretching exercise).	Both groups were seen by the nurse specialist at the baseline assessment.	HRQoL^b^ Mortality^a,b^ Walking test^b^ Dropout^a,b^	9 dropouts (5 in PR group and 4 in control group) at the end of treatment. 6 dropouts (2 in PR group and 4 in control group) at the longest follow-up.
Ko 2017 [33]	China	RCT	Setting: outpatient Duration: 8 weeks (1 year follow up) Frequency: 3/week	180 admitted patients with AECOPD (mean age: 75 years, FEV_1_: 42–47% of predicted, 94–97% men). Comorbidities: hypertension, type 2 diabetes, hyperlipidemia, ischemic heart disease, heart failure, old pulmonary tuberculosis.	PR consisted of education (smoking cessation, technique of using medications, nutrition, dyspnea management, self-management, psychological distress, exercise benefits and strategies, breathing and sputum-removal techniques) and individual physical training program to perform at home or a short course of outpatient PR.	Patients are offered supervised exercise training 3/week, if declining they are offered instructions for self-training, education, and telephone calls.	Usual care with medical treatment.	All patients received standard treatment with optimal medical therapy.	HRQoL^b^ Mortality^a^ Walking test^b^ Days in hospital^a^	38 dropouts (17 in PR group and 21 in control group)
Man 2004 [35]	England	RCT	Setting: outpatient Duration: 8 weeks Frequency: 2/week	42 admitted patients with AECOPD (mean age: 70 years, FEV_1_: 37–42% of predicted, 40% men). Comorbidities: not specified.	Supervised multidisciplinary PR, 1-h of exercise (aerobic walking and cycling, strength training for the upper and lower limb) and 1-h of education (with an emphasis on self-management of the disease, nutrition and lifestyle issues).	Supervised multidisciplinary PR.	Usual care with optimal medical treatment.	All admitted patients received standard treatment and home diaries which included a disease specific information pack.	HRQoF^b^ Mortality^b^ Walking test^b^ COPD related hospital readmissions^b^ Dropout^a^	8 dropouts (3 in PR group and 5 in control group)
Murphy 2005 [39]	Ireland	RCT	Setting: outpatient home-based Duration: 6 weeks Frequency: 2/week	31 admitted patients with AECOPD (mean age: 65–67 years, FEV_1_: 38–42% of predicted, 65% men). Comorbidities: not specified.	PR consisted of 30–40-min supervised home-based exercise program, aerobic exercises including stepping up and down a stair, sitting to stand from a chair, upper limb strength exercises with low-impact elastic band at 3–5 on the Borg breathlessness score.	Supervised home-based exercise program.	Standard medical treatment without any form of PR exercises or lifestyle changes advice.	All patients received standard medical treatment.	Walking test^a^ COPD related hospital readmissions^b^ Dropout^a^	5 dropouts (3 in PR group and 2 in control group)
Puhan 2012 [30]	Switzerland	RCT	Setting: in- and outpatient Duration: 12 weeks Frequency: 24 sessions (range 18–36)	36 admitted patients with AECOPD (mean age: 67 years, FEV_1_: 43–46% of predicted, 58% men). Comorbidities: cardiovascular, endocrine, musculoskeletal, other.	Early inpatient PR within 2 weeks after exacerbation, exercise training included endurance, strength and calisthenics training in addition with education (e.g. individual action plan, mediational use, exercise at home, coping with daily activities, smoking cessation).	Outpatient PR, exercise training included endurance, strength and calisthenics training in addition with education (as described under intervention).	Late PR starting 6 mo. after exacerbation, exercise training included endurance, strength and calisthenics training in addition with education.	Recommended number of exercise session 24 (ranged between 18 and 36).	Mortality^a^ Dropout^a^	8 dropouts (4 in PR group and 4 in control group)
Revitt 2018 [37]	United Kingdom	RCT	Setting: inpatient Duration: 6 weeks Frequency: 2/week	28 admitted patients with AECOPD (mean age: 66 years; FEV_1_: 1.18 l). Comorbidities: not specified.	Early PR within 4 weeks of discharge. PR consisted of individualized aerobic and resistance exercises and education on chest clearance and energy conservation.	Hospital-based PR.	Late PR initiated 7 weeks after discharge including exercise and education.	All patients received the same PR program.	Dropout^a^	11 dropouts (3 in control group prior to the program and 8 in PR group during the program)
Seymour 2010 [36]	United Kingdom	RCT	Setting: outpatient (hospital-led) Duration: 8 weeks Frequency: 2/week	60 admitted patients with AECOPD (mean age: 65-67 years, FEV_1_: 52% of predicted, 45% men). Comorbidities: hypertension, type 2 diabetes, ischemic heart disease.	PR consisted of supervised exercise training including a mixture of limb strengthening and aerobic activities tailored to individual baseline function and education session (lasting 2 h).	Hospital-led supervised exercise training.	Usual care with optimal medical treatment.	All patients were provided with general information about COPD and offered outpatient appointments with their general practitioner or respiratory team.	HRQoF^b^ Walking test^a^ COPD related hospital readmissions^b^ Dropout^b^	11 dropouts (7 in PR group and 4 in control group)
Troosters 2000 [38]	Belgium	RCT	Setting: outpatient Duration: 6 mo (18 mo follow up) Frequency: 2–3/week	100 patients with AECOPD referred to outpatient clinic (mean age: 60–63 years, FEV_1_: 41–43% of predicted, 87% men). Comorbidities: not specified.	PR consisted of 90-min supervised ET and RT. ET consisting of cycling, treadmill walking, and stair climbing at 60–80% of initial W_max_ during cycle ergometer/maximal walking speed. RT consisting of strength exercises for 5 muscle groups, 10 reps at 60% 1RM.	Supervised outpatient exercise training.	Usual medical care consisting of standard community care with respirologist.	During exercise training supplemental oxygenwas given to maintain oxygen saturation above 90%.	Mortality^a^ walking test^a^ dropout^a,b^	30 dropouts (13 in PR group and 17 in control group) at the end of treatment. 21 dropouts (11 in PR group and 10 in control group) at the longest follow-up.

*AECOPD* acute exacerbations of chronic obstructive pulmonary disease, *COPD* chronic obstructive pulmonary disease, *CT* combined training, *ET* endurance training, *FEV*_*1*_ forced expiratory volume in 1 s, *HR*_*max*_ maximum heart rate, *HRQoL* health related quality of life, *RCT* randomized controlled trial, *1RM* one repetition maximum, *RT* resistance training, *Reps* repetitions, *VO*_*2max*_ maximal oxygen uptake, *W*_*max*_ maximal work load in Watts, *6MWD* 6 min walking distance, *6MWT* 6 min walking test

^a^After end of treatment

^b^After longest follow up

### Risk of bias within studies

**Fig. 2:**
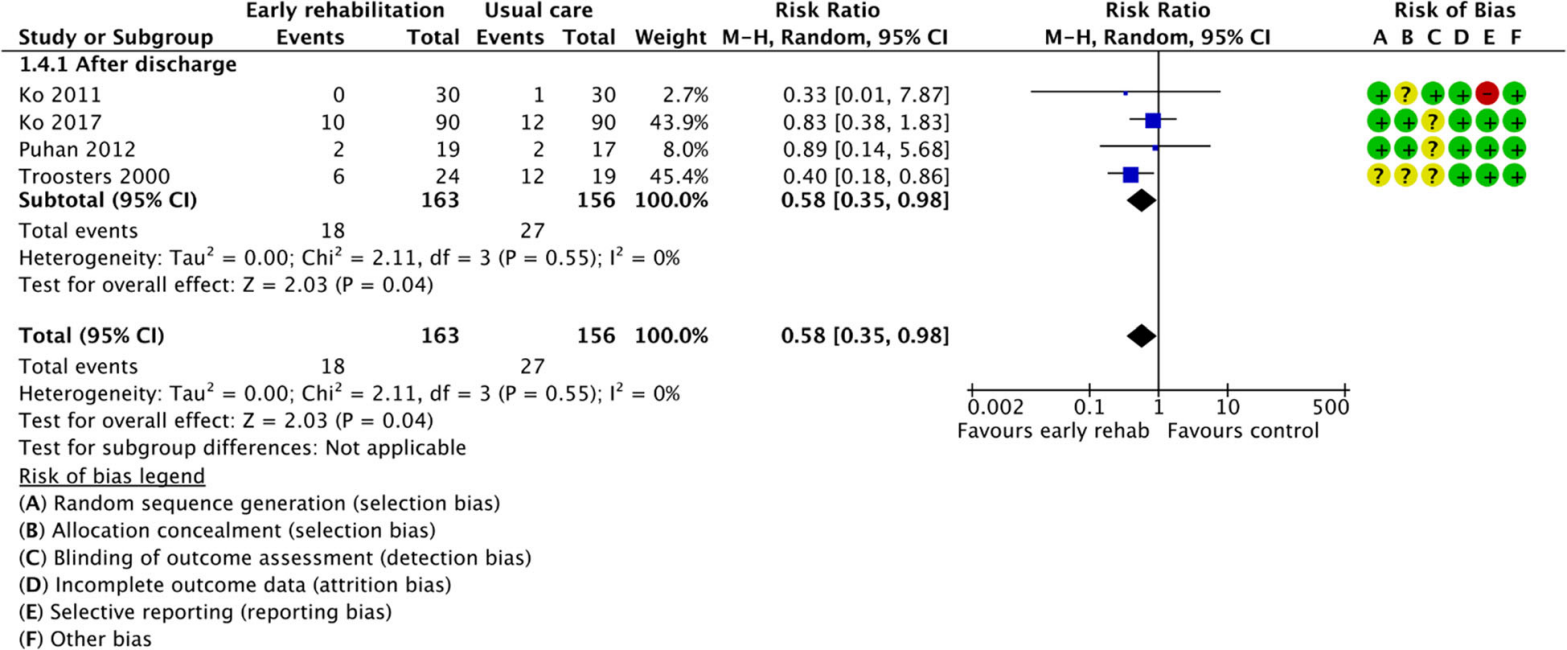
The effect of supervised early PR versus usual care on mortality at the end of treatment.

**Fig. 3:**
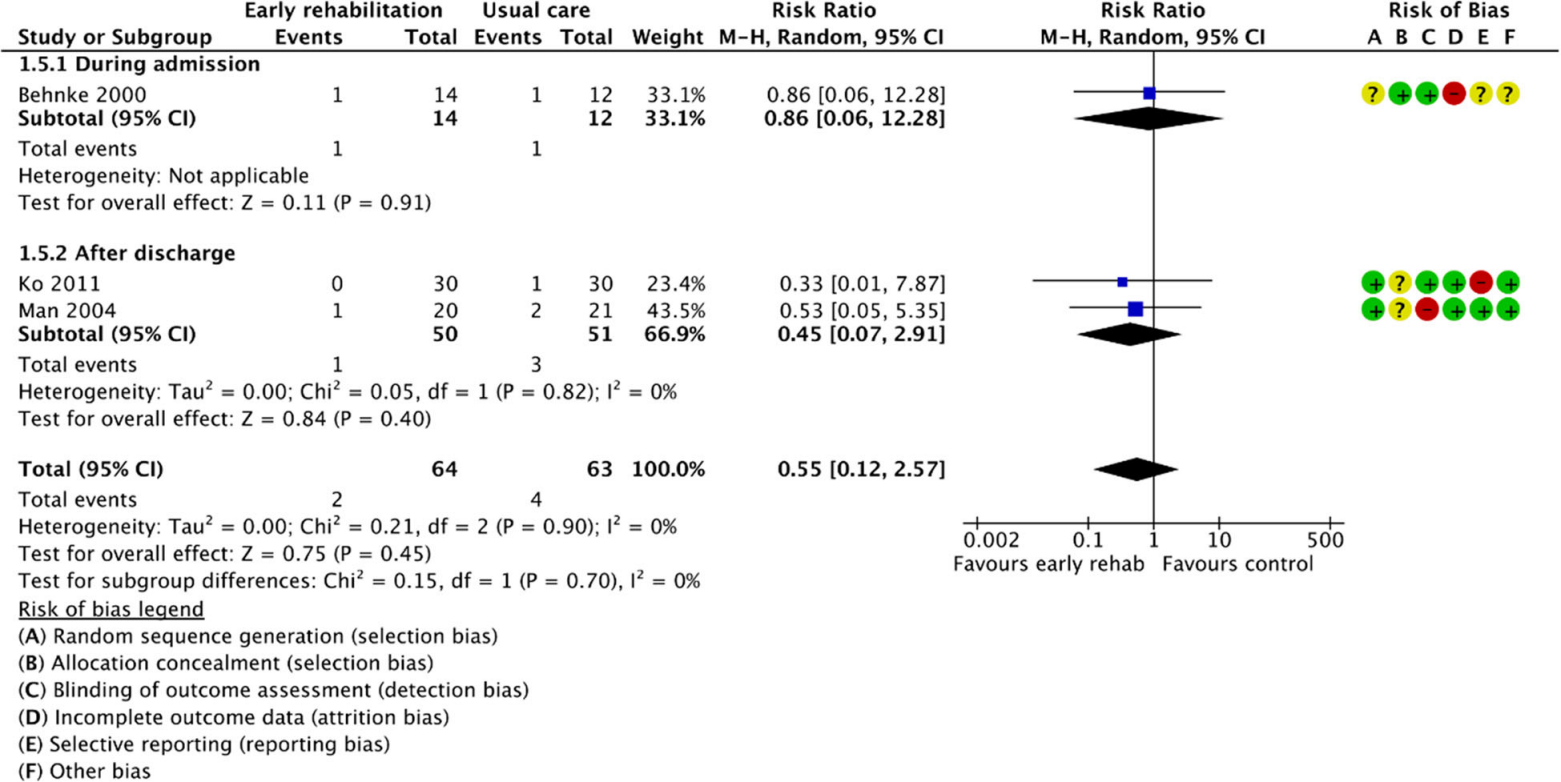
The effect of supervised early PR versus usual care on mortality at the longest follow up

**Fig. 4:**
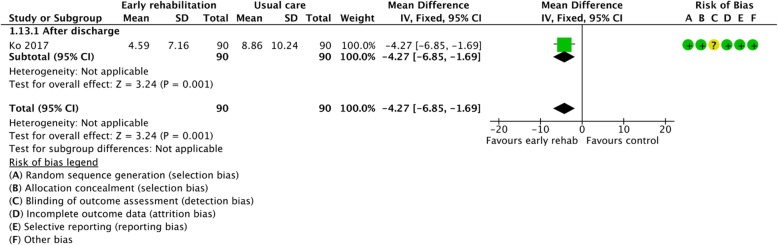
The effect of supervised early PR versus usual care on days in hospital at the end of treatment

**Fig. 5:**
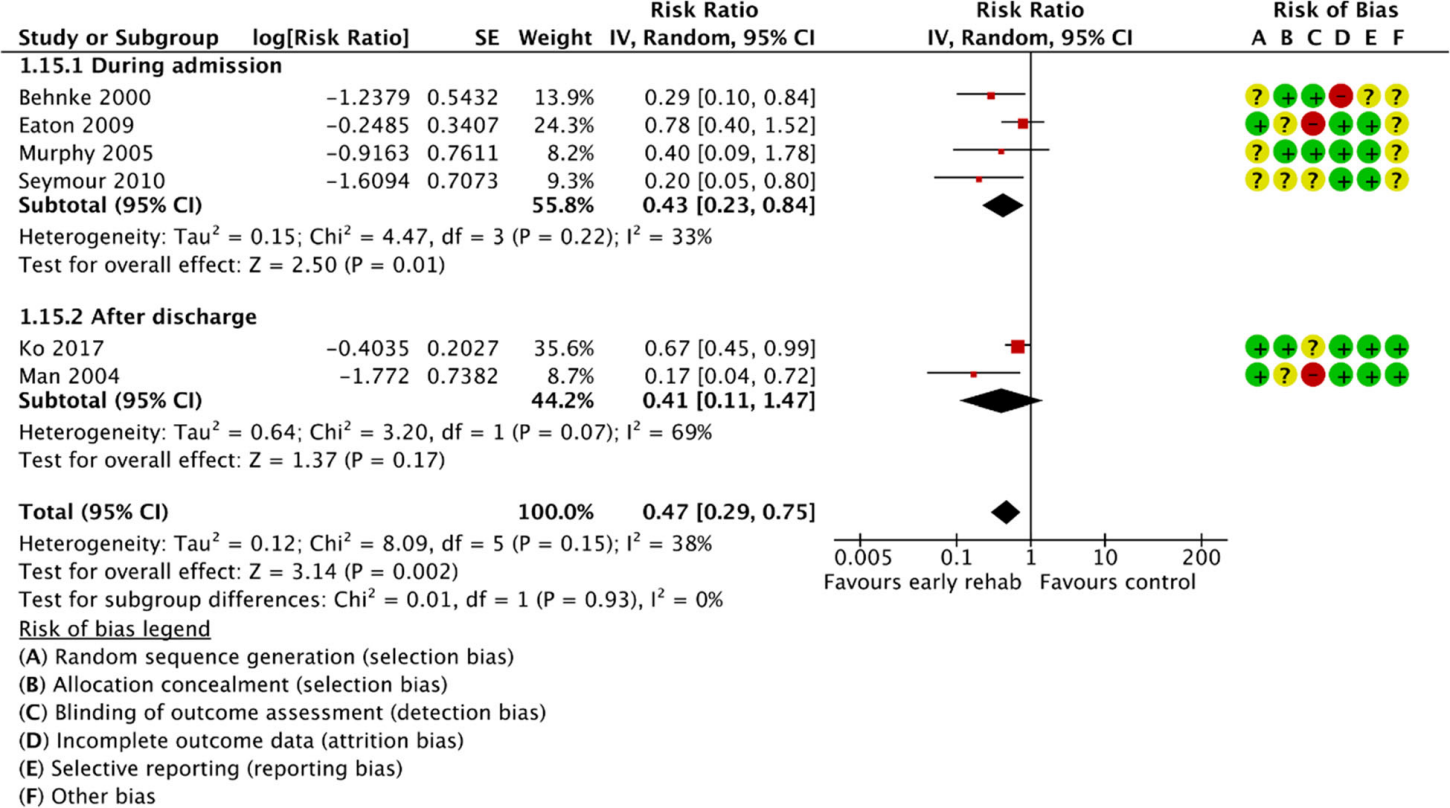
The effect of supervised early PR versus usual care on COPD related hospital readmissions at the longest follow up

**Fig. 6:**
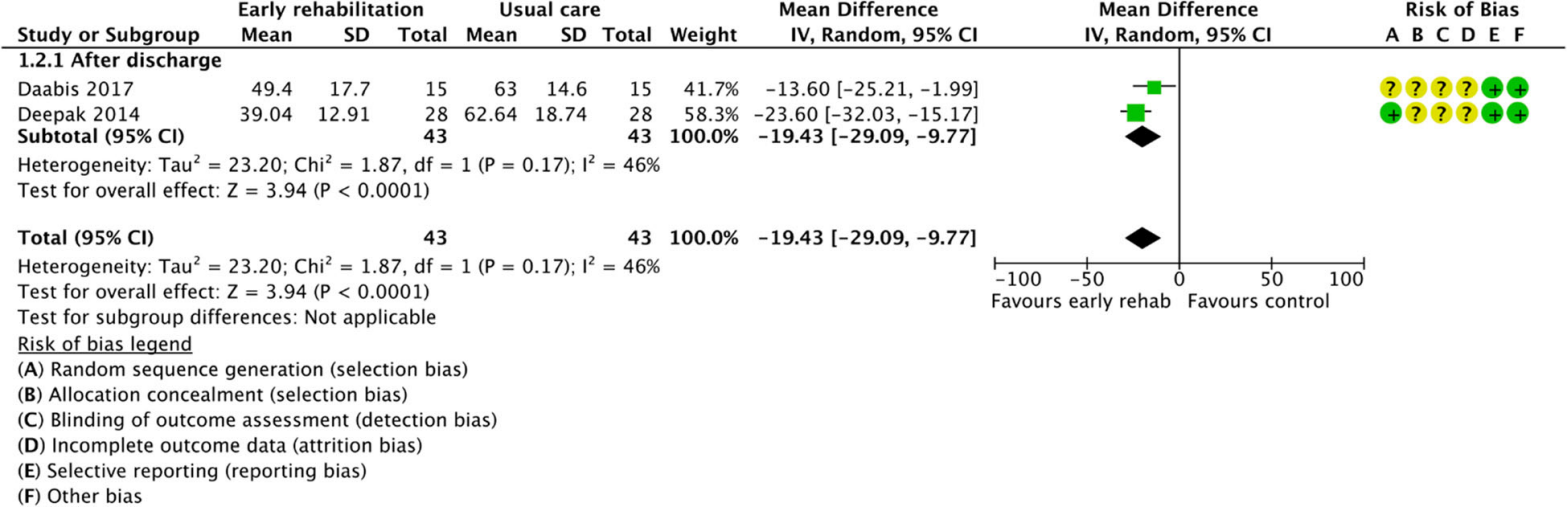
The effect of supervised early PR versus usual care on health-related quality of life at the end of treatment using the St. George’s Respiratory Questionnaire

**Fig. 7:**
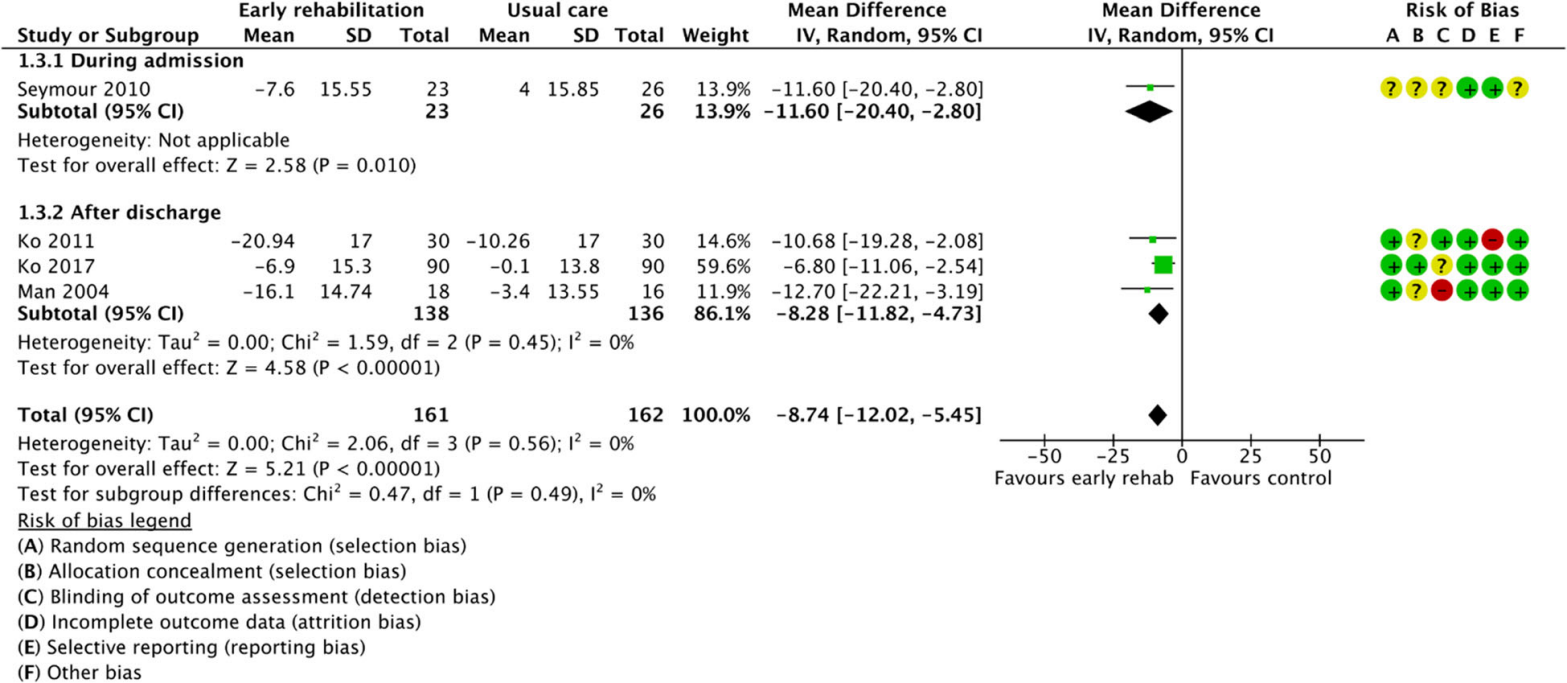
The effect of supervised early PR versus usual care on health-related quality of life at the longest follow up using the St. George’s Respiratory Questionnaire

**Fig. 8:**
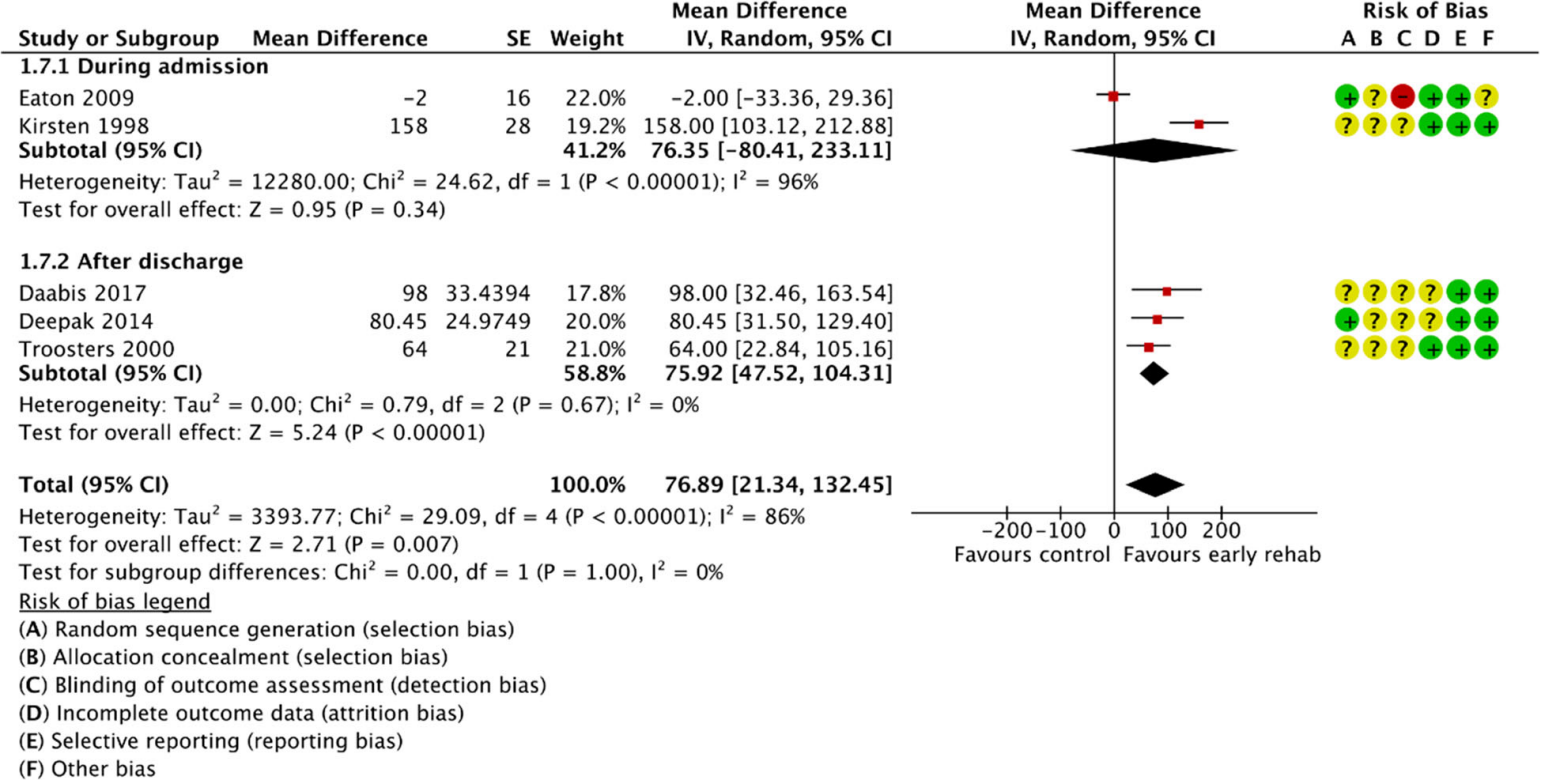
The effect of supervised early PR versus usual care on walking distance at the end of treatment using the 6-Minute Walking Test

**Fig. 9:**
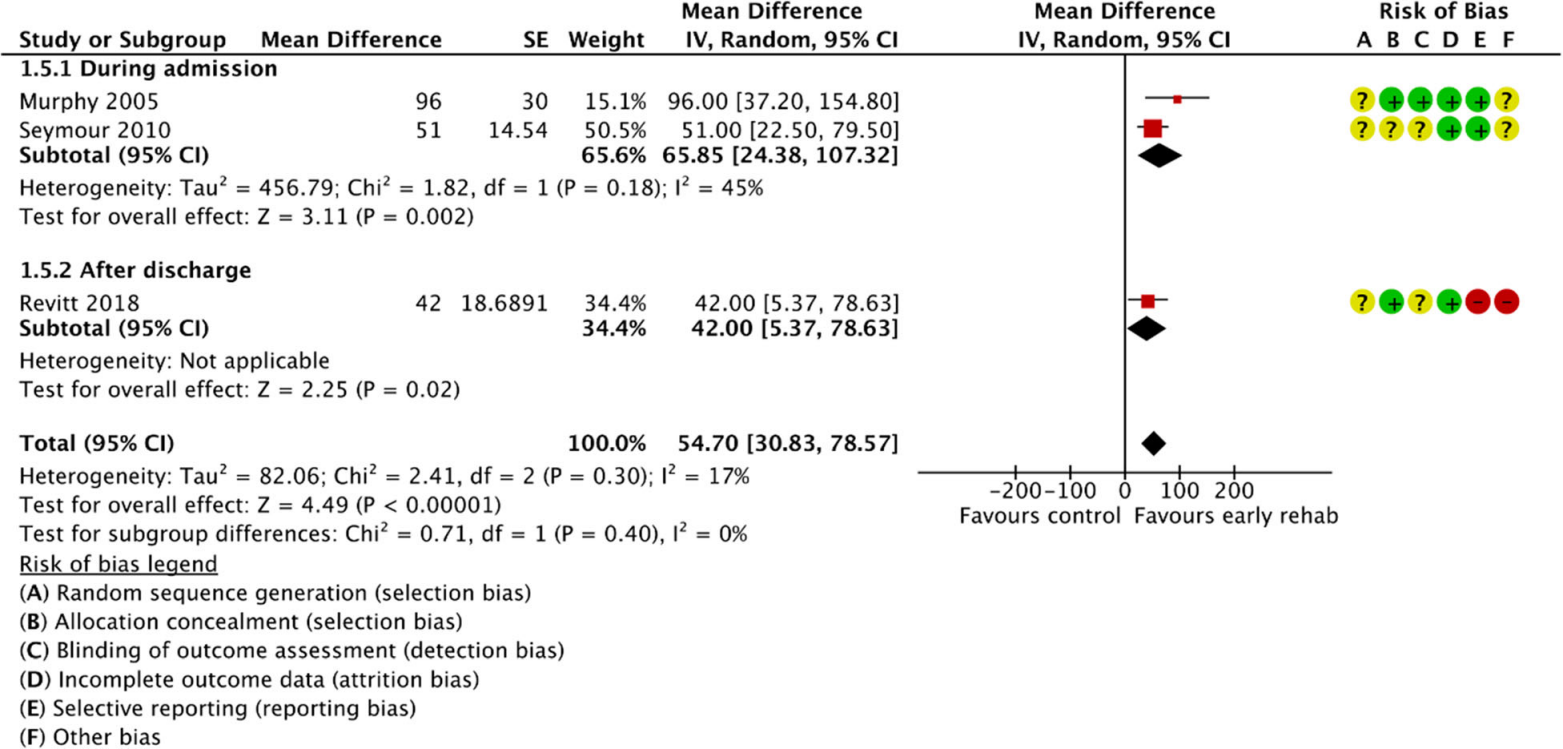
The effect of supervised early PR versus usual care on walking distance at the end of treatment using the Shuttle Walking Test

**Fig. 10:**
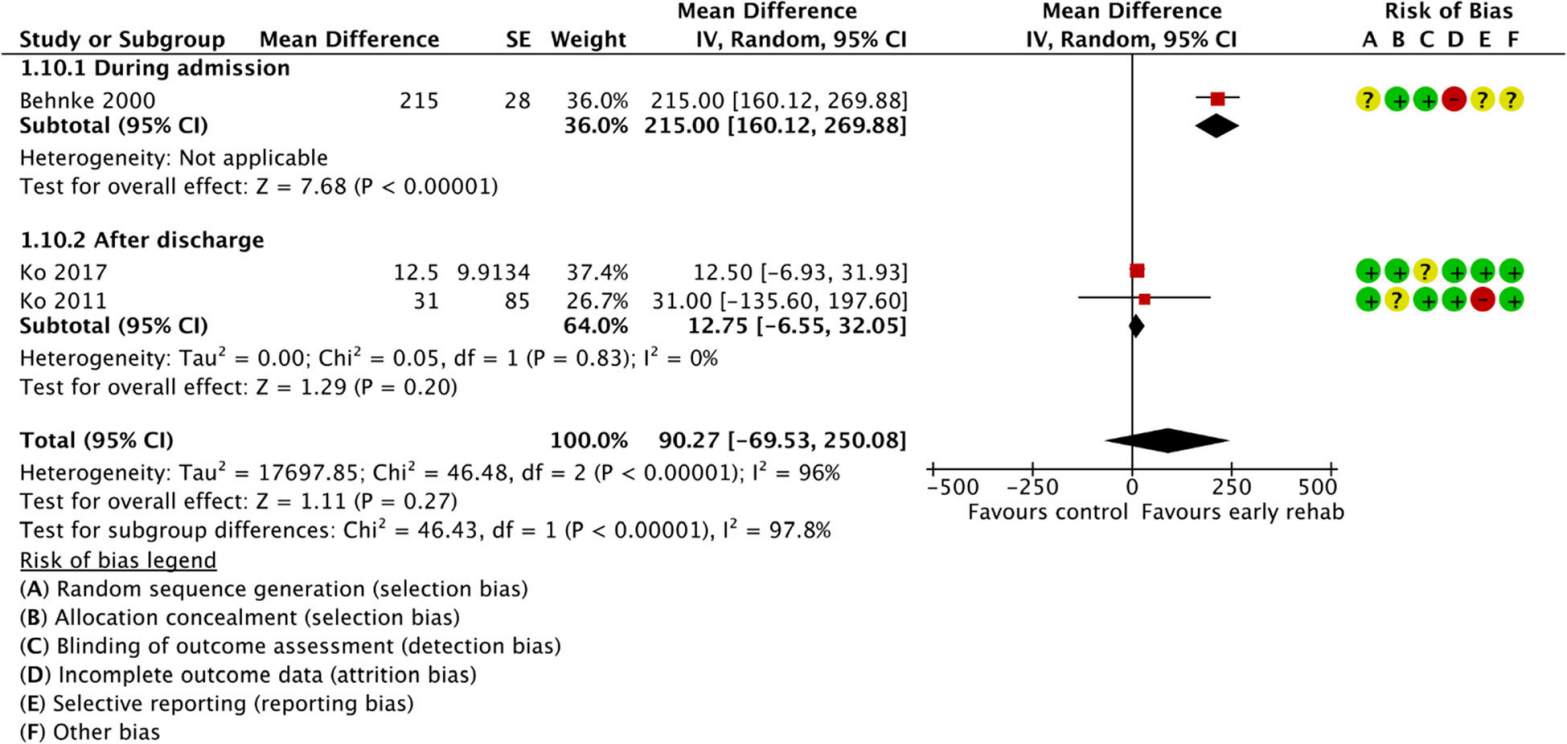
The effect of supervised early PR versus usual care on walking distance at the longest follow up using the 6-Minute Walking Test

**Fig. 11:**
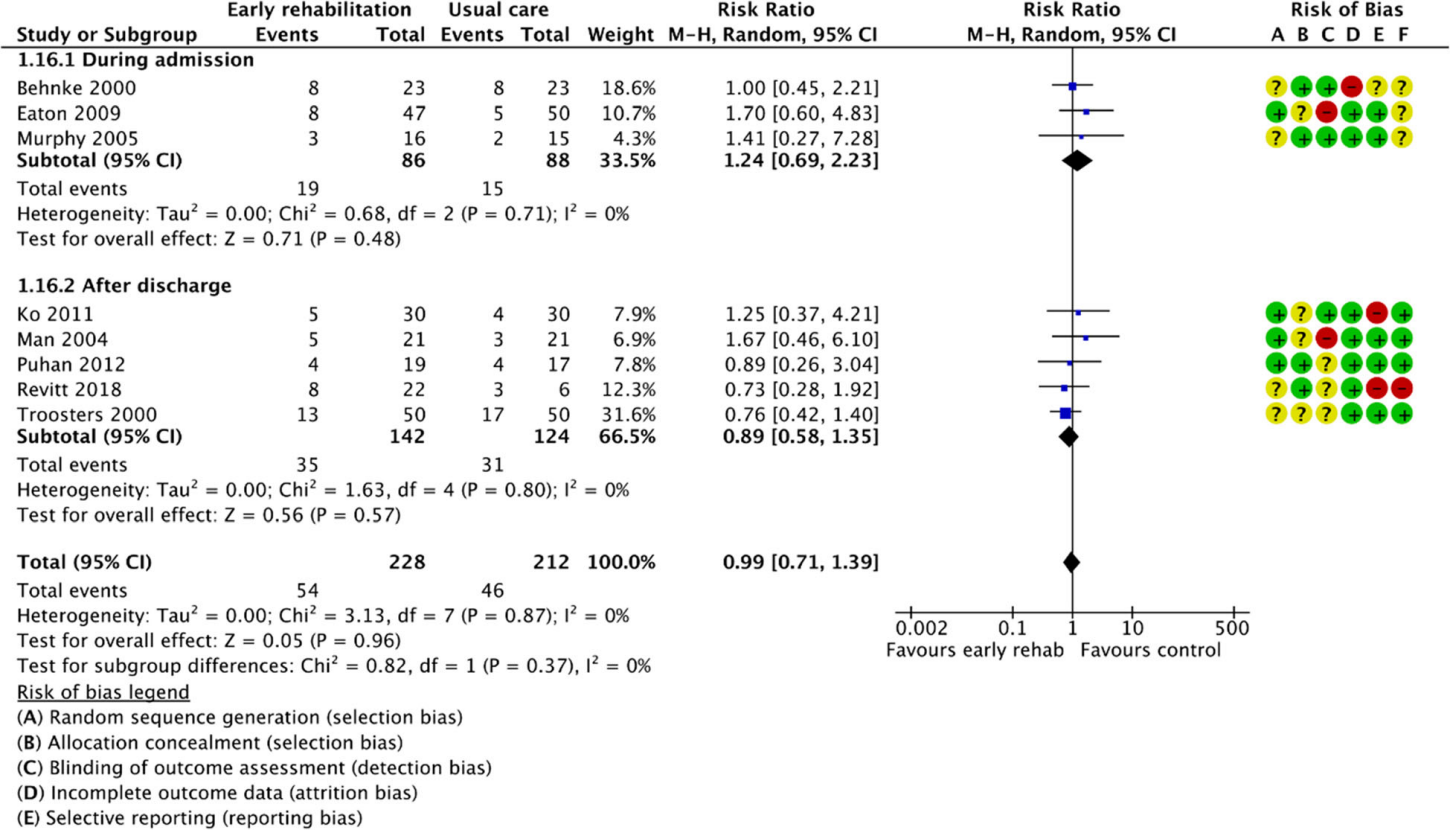
The effect of supervised early PR versus usual care on dropout at the end of treatment

**Fig. 12:**
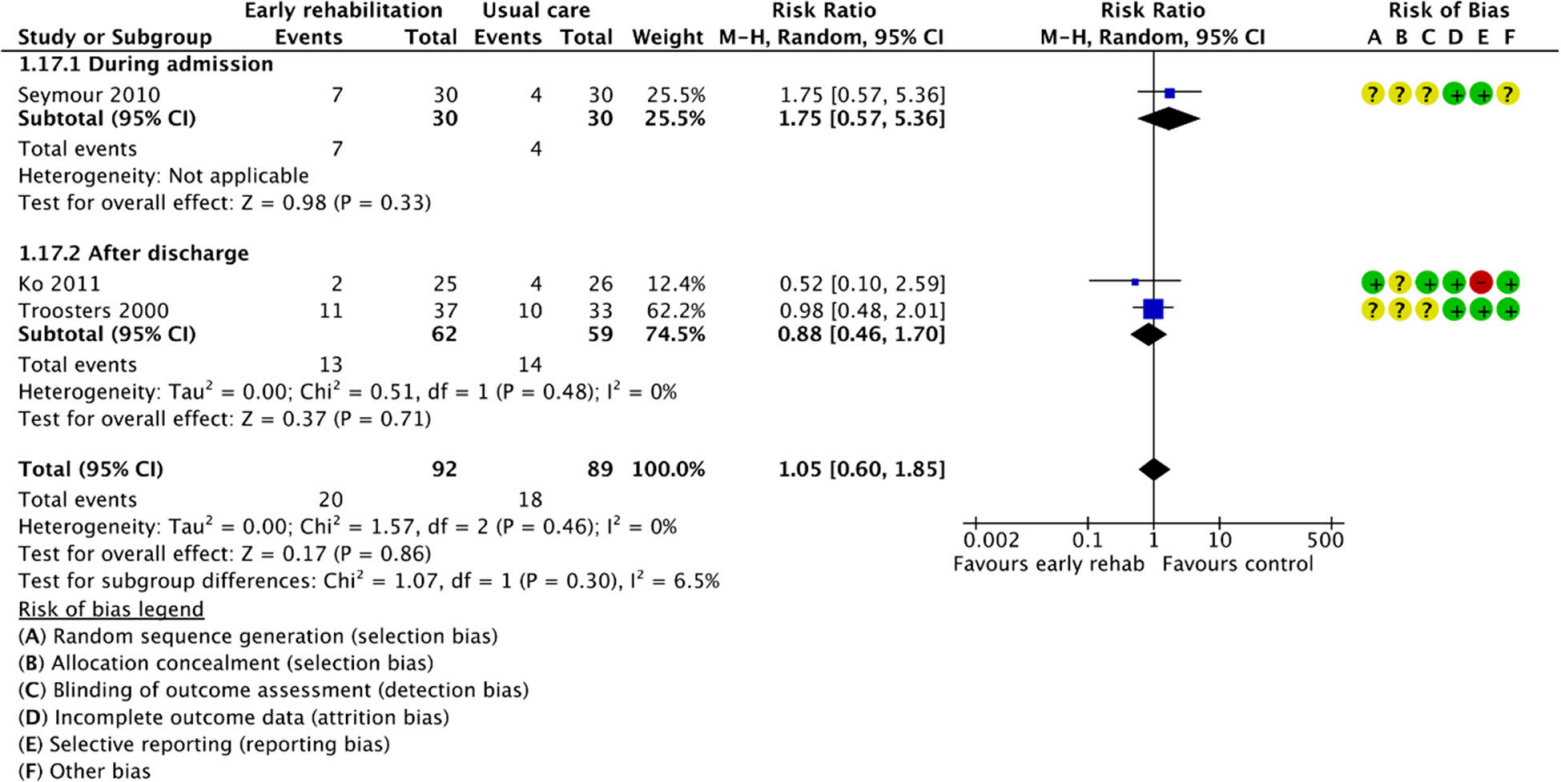
The effect of supervised early PR versus usual care on dropout at the longest follow up

Figures 2, 3, 4, 5, 6, 7, 8, 9, 10, 11 and 12 and Additional file 4 shows risk of bias of the included studies. In nine studies [28, 29, 31, 32, 34–38] the allocation concealment was not described, while seven studies [27, 29, 31, 36–39] did not report the randomization process. Three studies [27, 34, 39] blinded the personnel, with only two of the studies [34, 39] blinding the outcome assessor. One study [27] was assessed as having a high risk of incomplete outcome data reporting due to a large dropout. Selective outcome reporting of outcome measures was detected in one study [34]. No other sources of bias were detected. Thus, the quality of evidence from all studies included was downgraded due to risk of bias (Table 2, Additional file 4).

**Table 2:** GRADE Evidence Profile

Supervised early PR versus usual care for patients with acute exacerbation of COPD					
Outcome Timeframe	Study results and measurements	Absolute effect estimates		Certainty in the effects estimates (Quality of evidence)	Plain text summary
		Usual care	Early PR		
Mortality End of treatment Critical	Relative risk 0.58 (CI 95% 0.35–0.98) Based on data from 319 patients (4 studies)	173 per 1.000	100 per 1.000	Moderate Due to serious risk of bias^a^	Early pulmonary rehabilitation probably decreases mortality at the end of treatment
		Difference: 73 fewer per 1.000 (CI 95% 112 fewer - 3 fewer)			
Mortality Longest follow-up Critical	Relative risk 0.55 (CI 95% 0.12–2.57) Based on data from 127 patients (3 studies)	63 per 1.000	35 per 1.000	Low Due to serious risk of biasand serious risk of imprecision^a,b^	Early pulmonary rehabilitation may decrease mortality slightly at the longest follow-up
		Difference: 28 fewer per 1.000 (CI 95% 55 fewer - 99 more)			
Days in hospital End of treatment Important	Measured by: Days Lower is better Based on data from 180 patients (1 study)	0.86 (mean)	4.59 (mean)	Moderate Due to serious imprecision^c^	Early pulmonary rehabilitation probably decreases days in hospital at the end of treatment
		Difference: MD 4.27 lower (CI 95% 6.85 lower - 1.69 lower)			
Days in hospital Longest follow-up Important					No studies were found that looked at number of days in hospital at the longest follow-up
Readmission due to exacerbation End of treatment Important					No studies were found that looked at readmission to hospital due to exacerbation at the end of treatment
Readmission due to exacerbation Longest follow-up Important	Rate ratio 0.47 (CI 95% 0.29–0.75) Based on data from 365 patients (6 studies)			Moderate Due to serious risk of bias^a,d^	Early pulmonary rehabilitation probably decreases readmission to hospital due to exacerbation at the longest follow-up
Health-related quality of life End of treatment Important	Measured by: SGRQ Lower is better Based on data from 86 patients (2 studies)	Difference: MD 19.43 lower (CI 95% 29.09 lower - 9.77 lower)		Low Due to serious risk of bias and serious risk of imprecision^a,c^	Early pulmonary rehabilitation may improve health-related quality of life at the end of treatment
Health-related quality of life Longest follow-up Important	Measured by: SGRQ Lower is better Based on data from 323 patients (4 studies)	Difference: MD 8.74 lower (CI 95% 12.02 lower - 5.45 lower)		Moderate Due to serious risk of bias^a,d^	Early pulmonary rehabilitation probably improves health-related quality of life at the longest follow-up
Exercise capacity End of treatment Important	Measured by: SWT (meters) Higher is better Based on data from 95 patients (3 studies)	Difference: MD 54.7 more (CI 95% 30.83 more - 78.57 more)		Moderate Due to serious risk of bias^a,d^	Early pulmonary rehabilitation probably increases exercise capacity at the end of treatment
Exercise capacity End of treatment Important	Measured by: 6MWT (meters) Higher is better Based on data from 274 patients (5 studies	Difference: MD 76.89 more (CI 95% 21.34 more - 132.45 more)		Low Due to serious risk of bias and serious inconsistency^a,d,e^	Early pulmonary rehabilitation probably increases exercise capacity at the end of treatment
Exercise capacity Longest follow-up Important	Measured by: SWT (meters) Higher is better Based on data from 2017 patients (3 studies)	Difference: MD 90.27 higher (CI 95% 69.53 lower - 250.08 higher)		Low Due to serious risk of bias and serious inconsistency leading to serious imprecision^a,b,d,e^	Early pulmonary rehabilitation may increase exercise capacity at the longest follow-up
Dropout rate End of treatment Important	Relative risk 0.99 (CI 95% 0.71–1.39) Based on data from 440 patients (8 studies)	217 per 1.000	215 per 1.000	Moderate Due to serious risk of bias^a,d^	Early pulmonary rehabilitation probably has little impact on the dropout rate at the end of treatment
		Difference: 2 fewer per 1.000 (CI 95% 63 fewer - 85 more)			
Dropout rate Longest follow-up Important	Relative risk 1.05 (CI 95% 0.6–1.85) Based on data from 181 patients (3 studies)	202 per 1.000	212 per 1.000	Moderate Due to serious risk of bias^a,d^	Early pulmonary rehabilitation probably has little impact on dropout at the longest follow-up
		Difference: 10 more per 1.000 (CI 95% 81 fewer - 172 more)			
Falls Longest follow-up Important					No studies were found that looked at falls at the longest follow-up
Activities of daily living End of treatment Important					No studies were found that looked at activities of daily living at the end of treatment
Activities of daily living Longest-follow-up Important					No studies were found that looked at activities of daily living at the longest follow-up

*CI* confidence interval, *COPD* chronic obstructive pulmonary disease, *MD* middle difference, *PR* pulmonary rehabilitation, *SGRQ* St. George’s Respiratory Questionnaire, *SWT* Shuttle Walking Test, *6MWT* 6 min walking test

Quality of evidence. High quality: We are very confident that the true effect lies close to that of the estimate of the effect; Moderate quality: We are moderately confident in the effect estimate, the true effect is likely to be close to the estimate of the effect, but there is a possibility that it is substantially different; Low quality: Our confidence in the effect estimate is limited, the true effect may be substantially different from the estimate of the effect

^a^Risk of bias: Serious. Unclear/inadequate sequence generation and unclear/inadequate concealment of allocation during randomization process resulting in potential for selection bias

^b^Risk of imprecision: Serious. Wide confidence intervals

^c^Risk of imprecision: Serious. Low number of patients

^d^Risk of bias: Serious. Inadequate/unclear or lack of blinding of outcome assessors resulting in potential for detection bias

^e^Risk of inconsistency: Serious. The magnitude of statistical heterogeneity was high

## Effect of the intervention

We preformed meta-analyses in ten of our predefined outcomes. Subgroup analyses were undertaken in order to reveal differences between PR initiated during admission or within one week after discharge and PR initiated between one and four weeks after discharge from hospital. For an overview of all the outcomes, our confidence in the estimates and our interpretations see Table 2 GRADE Evidence profile.

### Mortality

Total mortality after end of treatment was reported in four of the included studies, including 319 randomized participants (early PR: *N* = 163; usual care: *N* = 156) [29, 32, 33, 37]. A total of 18 events were reported in the early PR group, whereas 27 events were reported in the usual care group. We found a statistically significant reduction in mortality favoring PR (RR = 0.58 (95% CI: [0.35 to 0.98])), with low heterogeneity (Fig. 2). The quality of evidence was downgraded due to unclear sequence generation, allocation concealment and blinding together with selective outcome reporting (Table 2).

Total mortality at longest follow up was reported in three of the included studies, including 127 participants (early PR: *N* = 64; usual care: *N* = 63) [26, 33, 34]. Two events were reported in the early PR groups while four events were reported in the usual care group. We found no statistical significant difference between groups (RR = 0.55 (95% CI: [0.12 to 2.57])). Subgroup analysis showed no difference in effect between trials with PR initiated during admission and after discharge (*P* = 0.70) (Fig. 3). Our confidence in the effect estimate was downgraded due to unclear sequence generation and allocation concealment together with lack of precision, incomplete outcome data and selective reporting (Table 2).

### Days in hospital

One study investigated the effect of early PR on the number of days in hospital after the end of treatment and stated that early PR led to a statistically reduction of 4.27 days (95% CI: [− 6.85 to − 1.69]) in the number of days in hospitals (Fig. 4). Accordingly, our confidence in the effect estimate was downgraded due to inclusion of only one study (Table 2).

### COPD related hospital readmissions

Six studies provided data from 365 participants on the number of COPD related hospital readmissions 3–12 months from baseline [27, 28, 33, 35, 36, 38]. The pooled effect estimate showed a decrease in the number of COPD related hospital readmissions favoring the early PR (RR = 0.47 (95% CI: [0.29 to 0.75])). Low heterogeneity (I^2^ = 38%) was observed, and the subgroup analysis showed no difference in effect between trials with PR initiated during admission and after discharge (*P* = 0.93) (Fig. 5). The quality of evidence was downgraded due to unclear sequence generation and allocation concealment together with lack of blinding and incomplete outcome date (Table 2).

### Health-related quality of life

The St. George’s Respiratory Questionnaire (SGRQ) (scale from 0 to 100, lower is better) were used across studies to assess HRQoL. Two studies were included and data from 86 participants were pooled in a meta-analysis evaluating HRQoL directly after end of early PR [31, 32] and showed a statistically and clinically significant improvement of 19.43 units on the SGRQ scale (95% CI: [− 29.09 to − 9.77]) in the early PR group compared with the usual care group (Fig. 6) with low heterogeneity. Our confidence in the effect estimate was downgraded due to unclear sequence generation, allocation concealment, blinding of assessors and incomplete outcome data (Table 2).

Four different studies provided data from 323 participants on the effect of early PR on HRQoL 3–12 months from baseline [33–36] and showed a statistically and clinically relevant improvement of 8.74 units on the SGRQ scale (95% CI: [− 12.02 to − 5.45]) in the early PR group compared with the usual care group (Fig. 7). Subgroup analysis showed no difference in effect between trials with PR initiated during admission and after discharge (*P* = 0.49). Unclear sequence generation, allocation concealment, blinding and selective outcome reporting led to downgrading of the confidence in our effect estimates (Table 2).

### Walking distance

The walking distance (6-Minute Walking Test (6MWT) or Shuttle Walking Test (SWT)) after the end of treatment was investigated in eight studies [28, 29, 31, 32, 36–39]. Pooling the results (early PR: *N* = 139; usual care: *N* = 135) from five trials using 6MWT yielded a statistically significant mean difference in walking distance of 76.89 m, favoring early PR (95% CI: [21.34 to 132.45]) with high heterogeneity (Fig. 8). The subgroup analysis showed no difference in the effect between PR initiated during admission and after discharge (*P* = 1.00). However, we found a significant within-group effect of early PR after discharge (Fig. 8). The quality of evidence was downgraded due to unclear sequence generation, allocation concealment, blinding of assessors and incomplete data together with high risk of inconsistency (Table 2). Three trials (early PR: *N* = 50; usual care: *N* = 45) used the SWT to evaluate the walking distance after the end of treatment and showed a statistically significant mean difference in walking distance of 54.70 m, favoring early PR (95% CI: [30.83 to 78.57]). The subgroup analysis showed no difference in the effect between PR initiated during admission and after discharge (*P* = 0.40). However, we found a significant within-group effect of early PR during admission and after discharge (Fig. 9). The quality of evidence was downgraded due to unclear sequence generation, allocation concealment, blinding of assessors, incomplete outcome together with selective outcome reporting (Table 2).

Three different studies provided data from 217 participants on the effect of early PR on walking distance assessed by 6MWT at 3–12 months from baseline [27, 33, 34] and showed no statistically, but a clinically relevant difference (mean difference: 90.27 m; 95% CI: [− 69.53 to 250.08]) with high heterogeneity (Fig. 10). Subgroup analysis showed a statistically significant difference between groups in favor of early PR during admission (*P* < 0.01) (Fig. 10). Due to unclear sequence generation, allocation concealment, blinding of assessors, incomplete data and selective reporting together with high risk of inconsistency leading to high risk of imprecision the quality of evidence was downgraded (Table 2).

### Drop-outs

The effect of early PR on the drop-out rate at the end of treatment was investigated in eight studies providing data from 440 randomized participants (early PR: *N* = 228; usual care: *N* = 212) [27, 28, 30, 34, 35, 37–39]. A total of 54 drop-outs were reported in the early PR group, whereas 46 drop-outs were reported in the usual care group, with no significant difference between groups (RR = 0.99 (95% CI: [0.71 to 1.39])) (Fig. 11). The subgroup analysis showed no difference in the effect between PR initiated during admission and after discharge (*P* = 0.37). Our confidence in the effect estimate was downgraded due to unclear sequence generation, allocation concealment, blinding of assessors and incomplete data outcome together with high risk of inconsistency (Table 2).

Three different studies provided data from 181 participants on the effect of early PR on drop-out at 3–18 months from baseline (early PR: *N* = 92; usual care: *N* = 89) [34, 36, 38]. A total of 20 drop-outs were reported in the early PR group, while 18 drop-outs were reported in the usual care group, with no difference between groups (RR = 1.05 (95% CI: [0.60 to 1.85])) (Fig. 12, Table 2). Subgroup analysis showed no difference in the effect between trials with PR initiated during admission and after discharge (*P* = 0.30; I^2^ = 6.5%) (Fig. 12).

None of the included studies reported results on the effect of early PR on ADL or the risk of falling.

## Discussion

### Summary of main findings

The present review summarized the evidence from 13 RCTs including 634 participants with an exacerbation of COPD and compared the use of early PR (*N* = 322) with usual care (*N* = 312). Subsequent meta-analysis showed that supervised early PR after acute exacerbation of COPD reduced mortality and number of days in hospital together with a reduction in COPD related hospital admissions and an improvement of HRQoL and exercise capacity (walking distance).

### Mortality

We found that supervised early PR in patients with exacerbation of COPD reduced risk of mortality by ~ 42% compared with usual care. This finding was based on moderate quality of evidence due to methodological issues in the included studies and the relatively small numbers of participants. While similar conclusions have been reported in guidelines and systematic reviews in the past, results from a resent RCT by Greening et al. questioned the beneficial effects by reporting higher mortality in the early PR group [15–17]. In this study authors included patients with COPD related exacerbations during admission and instructed participants in the intervention group to be more physical active the next three months facilitated by technical devices [17]. In contrast, the majority of evidence favoring PR in stable COPD is based on supervised programs, and therefore we did not include Greening et al. in our review. However, to assess safety of early PR initiated during the hospital admission we performed a subgroup analysis showing no difference between groups rehabilitated during the admission and after discharge.

Results from this review differ from a previous review by Puhan et al. [14] who showed no statistically significant effect of early PR on mortality, but when the authors preformed a subgroup analysis, excluding results from Greening et al. [17], they did find a positive effect of early PR on mortality [14]. Moreover, the review by Puhan et al. [14] differs methodologically from the present review, as they included any inpatient and/or outpatient PR program with no criteria for the comprehensiveness or supervision of the rehabilitation program. We only included studies of supervised PR programs similar to what is offered to COPD patients in Denmark, which is based on the present large amount of evidence in favor of supervised PR in stable COPD. This might explain the lower heterogeneity and greater effects on mortality in the present review.

### Hospital length of stay and readmissions

Moderate-quality evidence showed that supervised early PR significantly reduced the risk of COPD related hospital readmissions at the longest follow up with 53%. In addition, the number of days in hospital was reduced by an average of 4.27 days. Puhan et al. [14] have previously shown that PR significantly reduced the mean number of hospital admissions per participant from 1.6 to 0.9 during the year following after hospital admission for an acute exacerbation. Several explanations have been proposed for the substantial effect of PR on hospital readmission. The main reason is probably that hospitalization following an acute exacerbation of COPD leads to significant reductions in activity level [6]. It is well known that the recovery period after an acute exacerbation is long, even for patients with no subsequent exacerbations [40]. Thus, PR can be considered an effective intervention for reverting physical inactivity [41] and it has been shown that patients who achieved improvement in their daily physical activity level after an exacerbation of COPD experienced a ~ 50% reduction in risk of hospital readmission [42].

### Health-related quality of life and exercise capacity

The primary result to support this, in the present review, are clinically relevant improvements in walking distance of respectively 76.89 m in 6 min walking distance (6MWD) and 54.70 m in shuttle walking distance (SWD) immediately after early PR and an improvement of 90.27 m in 6MWD at the longest follow up [43], which are in line with those results from Puhan et al. [14], showing an improvement of 62.38 m in 6MWD after early PR. Secondly, we found moderate quality of evidence supporting a clinically important improvement in HRQoL immediately after participation of 19.43 units on the SGRQ scale and an improvement of 8.74 units at the longest follow up. These effects on HRQoL exceeded the minimal clinically important difference (MCID) for the SGRQ (> 4-point improvement [44]), and the results are in line with previous studies showing a large effect of PR on HRQoL in stable patients with COPD [14]. Although statistically non-significant, the beneficial effects of early PR versus usual care on SGRQ at the longest follow up (8.74 units) in present review were close to those observed in stable COPD patients (6.89 units) [9]. In addition, the present review found a greater improvement in HRQoL at the end of treatment in patients with an exacerbation of COPD compared with stable COPD patients, which probably is due to the lower baseline during recovery from AECOPD.

### Clinical application

We found no difference in drop-out rate between participants allocated to early PR compared with usual care. Thus, the effects were not driven solely by positive responders to PR, and secondly, the most severely affected patients will likely complete or drop-out to the same extent as usual care. As before mentioned, we did not include Greening et al. [17], since this study has been highly criticized for not offering a sufficiently extensive PR programs [45, 46], and interestingly, authors reported a high number of drop-outs. The participants in the rehabilitation group attended an average of 2.6 supervised sessions during hospital admission, followed by mainly unsupervised training after discharge, with a poor adherence to the home self-management exercise program (mean of 57.5) [17]. Nevertheless, these results suggest that it is important to assess how the PR is delivered. PR programs can differ in many aspects, which may influence their effectiveness. When assessing the extensiveness of the PR program; the number of exercise training sessions, frequency of exercise training, type of exercise training and supervision of training, as well as self-management, education and adherence to the PR program need to be considered [26].

In this review ten studies implemented an extensive PR program which mostly showed large and consistent effects on mortality, days in hospital, COPD related hospital readmissions, HRQoL, and walking distance. The PR programs were not exactly similar within the reviewed studies, but the majority provided either many training sessions (more than 16 sessions) [27, 29–31, 33, 34, 38], programs of long duration (> 12 weeks) [27, 38], or supported education [28, 30, 33, 35, 36]. Nevertheless, our results show that supervised early PR programs across studies with different protocols are effective in patients with COPD-related exacerbations.

### Safety

Currently, the ideal timing of the onset of PR after AECOPD is highly debated. Based on low-quality of evidence, the ERS/ATS Task Force made a conditional recommendation against the initiation of PR during hospitalization since PR initiated during admission was found to increase mortality [18]. This conclusion seems based solely on results from Greening et al. [17]*,* who reported a higher mortality in the unsupervised home-based rehabilitation group at 12 months compared with usual care group. The difference between groups however, was not related to the early rehabilitation intervention. Indeed, the per protocol analysis did not show a difference in mortality [17], suggesting that the participants who actually received the intervention were not accountable for the increased mortality [47]. We did not find any harms of early supervised PR across 13 RCTs, even when we isolated the subgroup that initiated PR during admission.

## Conclusion

The results of the present review support the substantial and clinical important benefits of supervised early PR, indicating that this is an effective intervention with the purpose of reducing mortality following a hospitalization for AECOPD. Our meta-analysis shows that supervised PR during the recovery period after an AECOPD is superior to usual care in terms of improving prognosis, HRQoL and walking distance. Based on moderate to low quality of evidence, we conclude that supervised early PR reduces the risk of mortality, COPD-related hospital readmissions and the number of days in hospital, and lead to large and clinically relevant improvements in HRQoL and walking distance. Therefore, we recommend supervised PR to patients with COPD-related exacerbations. PR should be initiated during hospital admission or within 4 weeks after hospital discharge.

## Additional files

Additional file 1: Search strategy. The full search strategy from the systematic multidatabase literature search performed in 2013 and 2017. (PDF 214 kb)

Additional file 2: AGREE II. A critical group appraisal of: Pulmonary rehabilitation for patients with chronic pulmonary disease (COPD): an evidence-based analysis using the AGREE II Instrument. (PDF 54 kb)

Additional file 3: AMSTAR (A Measurement Tool to Asses Systematic Reviews). An assessment of the methodological quality of the included systematic reviews. (PDF 10 kb)

Additional file 4: Assessment of the included studies. Characteristics and risk of bias assessment of the included studies. (PDF 329 kb)

Additional file 5: **Table S1.** Extensiveness of the PR programs in the included studies. (PDF 23 kb)

## Abbreviations

1RM: One repetition maximum
6MDT: 6-min walking test
6MWD: 6-min walking distance
ADL: Activities of daily living
AECOPD: Acute exacerbation in chronic obstructive pulmonary disease
AGREE II: Appraisal of Guidelines for Research and Evaluation instrument version II
AMSTAR: A Measurement Tool to Asses Systematic Reviews
ATS: American Thoracic Society
BTS: British Thoracic Society
COPD: Chronic obstructive pulmonary disease
CT: Combined training
ERS: European Respiratory Society
ET: Endurance training
FEV_1_: Forced expiratory volume in 1 s
GRADE: Grading of Recommendations Assessment, Development and Evaluation
HR_max_: Maximum heart rate
HRQoL: Health-related quality of life
I^2^: I-square
MCID: Minimal clinically important difference
MD: Mean difference
PICO: Population, intervention, comparison and outcomes
PR: Pulmonary rehabilitation
RCT: Randomized controlled trials
Reps: Repetitions
RT: Resistance training
SGRQ: St. George’s Respiratory Questionnaire
SMD: Standardized mean difference
SWD: Shuttle walking distance
SWT: Shuttle walking test
VO_2max_: Maximal oxygen uptake
W_max_: Maximal work load in Watts

## References

[1] Rosenberg SR, Kalhan R, Mannino DM (2015). Epidemiology of chronic obstructive pulmonary disease: prevalence, morbidity, mortality, and risk factors. Semin Respir Crit Care Med.

[2] Halpin DM, Miravitlles M, Metzdorf N, Celli B (2017). Impact and prevention of severe exacerbations of COPD: a review of the evidence. Int J Chron Obstruct Pulmon Dis.

[3] Singanayagam A, Schembri A, Chalmers JD (2013). Predictors of mortality in hospitalized adults with acute exacerbation of chronic obstructive pulmonary disease. Ann Am Thorac Soc.

[4] Goto T, Faridi MK, Gibo K, Toh S, Hanania NA, Camargo CA (2017). Trends in 30-day readmission rates after COPD hospitalization, 2006-2012. Respir Med.

[5] Eriksen N, Vestbo J (2010). Management and survival of patients admitted with an exacerbation of COPD: comparison of two Danish patient cohorts. Clin Respir J.

[6] Pitta F, Troosters T, Probst VS, Spruit MA, Decramer M, Gosselink R (2006). Physical activity and hospitalization for exacerbation of COPD. Chest.

[7] Donaldson GC, Wilkinson TM, Hurst JR, Perera WR, Wedzicha JA (2005). Exacerbations and time spent outdoors in chronic obstructive pulmonary disease. Am J Respir Crit Care Med.

[8] Seidel D, Cheung A, Suh ES, Raste Y, Atakhorrami M, Spruit MA (2012). Physical inactivity and risk of hospitalisation for chronic obstructive pulmonary disease. Int J Tuberc Lung Dis.

[9] McCarthy B, Casey D, Devane D, Murphy K, Murphy E, Lacasse Y (2015). Pulmonary rehabilitation for chronic obstructive pulmonary disease. Cochrane Database Syst Rev.

[10] Spruit MA, Singh SJ, Garvey C, ZuWallack R, Nici L, Rochester C (2013). An official American Thoracic Society/European Respiratory Society statement: key concepts and advances in pulmonary rehabilitation. Am J Respir Crit Care Med.

[11] Iepsen UW, Jørgensen KJ, Ringbaek T, Hansen H, Skrubbeltrang C, Lange P (2015). A systematic review of resistance training versus endurance training in COPD. J Cardiopulm Rehabil Prev..

[12] Iepsen UW, Jørgensen KJ, Ringbæk T, Hansen H, Skrubbeltrang C, Lange P (2015). A combination of resistance and endurance training increases leg muscle strength in COPD: an evidence-based recommendation based on systematic review with meta-analyses. Chron Respir Dis..

[13] Ringbaek T, Brondum E, Martinez G, Thogersen J, Lange P (2010). Long-term effects of 1-year maintenance training on physical functioning and health status in patients with COPD: a randomized controlled study. J Cardiopulm Rehabil Prev.

[14] Puhan MA, Gimeno-Santos E, Cates CJ, Troosters T (2016). Pulmonary rehabilitation following exacerbations of chronic obstructive pulmonary disease. Cochrane Database Syst Rev.

[15] Puhan MA, Gimeno-Santos E, Scharplatz M, Troosters T, Walters EH, Steurer J (2011). Pulmonary rehabilitation following exacerbations of chronic obstructive pulmonary disease. Cochrane Database Syst Rev.

[16] National Institute for Health and Clinical Excellence: Guidance. Chronic Obstructive Pulmonary Disease: Management of Chronic Obstructive Pulmonary Disease in Adults in Primary and Secondary Care: National Clinical Guideline Centre; 2010.

[17] Greening NJ, Williams JEA, Hussain SF, Harvey-Dunstan TC, Bankart MJ, Chaplin EJ (2014). An early rehabilitation intervention to enhance recovery during hospital admission for an exacerbation of chronic respiratory disease: randomised controlled trial. BMJ.

[18] Wedzicha Jadwiga A., Miravitlles Marc, Hurst John R., Calverley Peter M.A., Albert Richard K., Anzueto Antonio, Criner Gerard J., Papi Alberto, Rabe Klaus F., Rigau David, Sliwinski Pawel, Tonia Thomy, Vestbo Jørgen, Wilson Kevin C., Krishnan Jerry A. (2017). Management of COPD exacerbations: a European Respiratory Society/American Thoracic Society guideline. European Respiratory Journal.

[19] The Grading of Recommendations Assessment, Development and Evaluation (GRADE) Working Group. [Online] http://www.gradeworkinggroup.org. Accessed 28 Nov 2017.

[20] Guyatt GH, Oxman AD, Kunz R, Atkins D, Brozek J, Vist G (2011). GRADE guidelines: 2. Framing the question and deciding on important outcomes. J Clin Epidemiol.

[21] COPD Working Group (2012). Pulmonary rehabilitation for patients with chronic pulmonary disease (COPD): an evidence-based analysis. Ont Health Technol Assess Ser.

[22] Higgins JPT, Green S (editors). Cochrane Handbook for Systematic Reviews of Interventions Version 5.1.0 [updated March 2011]. The Cochrane Collaboration. 2011. Availble from http://handbook.cochrane.org. Accessed 28 Nov 2017.

[23] Review Manager (RevMan) [Computer program] (2014). Version 5.3.

[24] Guyatt GH, Oxman AD, Kunz R, Woodcock J, Brozek J, Helfand M (2011). GRADE guidelines: 7. Rating the quality of evidence--inconsistency. J Clin Epidemiol.

[25] Balshem H, Helfand M, Schünemann HJ, Oxman AD, Kunz R, Brozek J (2011). GRADE guidelines: 3. Rating the quality of evidence. J Clin Epidemiol.

[26] Bolton CE, Bevan-Smith EF, Blakey JD, Crowe P, Elkin SL, Garrod R (2013). British Thoracic Society guideline on pulmonary rehabilitation in adults. Thorax.

[27] Behnke M, Taube C, Kirsten D, Lehnigk B, Jörres RA, Magnussen H (2000). Home-based exercise is capable of preserving hospital-based improvements in severe chronic obstructive pulmonary disease. Respir Med.

[28] Eaton T, Young P, Fergusson W, Moodie L, Zeng I, O'Kane F (2009). Does early pulmonary rehabilitation reduce acute health-care utilization in COPD patients admitted with an exacerbation? A randomized controlled study. Respirology.

[29] Kirsten DK, Taube C, Lehnigk B, Jörres RA, Magnussen H (1998). Exercise training improves recovery in patients with COPD after an acute exacerbation. Respir Med.

[30] Puhan MA, Spaar A, Frey M, Turk A, Brändli O, Ritscher D (2012). Early versus late pulmonary rehabilitation in chronic obstructive pulmonary disease patients with acute exacerbations: a randomized trial. Respiration.

[31] Daabis R, Hassan M, Zidan M (2017). Endurance and strength training in pulmonary rehabilitation for COPD patients. Egypt J Chest Dis Tuberc.

[32] Deepak TH, Mohapatra PR, Janmeja AK, Sood P, Gupta M (2014). Outcome of pulmonary rehabilitation in patients after acute exacerbation of chronic obstructive pulmonary disease. Indian J Chest Dis Allied Sci.

[33] Ko FW, Cheung NK, Rainer TH, Lum C, Wong I, Hui DS (2017). Comprehensive care programme for patients with chronic obstructive pulmonary disease: a randomised controlled trial. Thorax.

[34] Ko FW, Dai DL, Ngai J, Tung A, Ng S, Lai K (2011). Effect of early pulmonary rehabilitation on health care utilization and health status in patients hospitalized with acute exacerbations of COPD. Respirology.

[35] Man WD, Polkey MI, Donaldson N, Gray BJ, Moxham J (2004). Community pulmonary rehabilitation after hospitalisation for acute exacerbations of chronic obstructive pulmonary disease: randomised controlled study. BMJ.

[36] Seymour JM, Moore L, Jolley CJ, Ward K, Creasey J, Steier JS (2010). Outpatient pulmonary rehabilitation following acute exacerbations of COPD. Thorax.

[37] Revitt Olivia, Sewell Louise, Singh Sally (2018). Early versus delayed pulmonary rehabilitation: A randomized controlled trial – Can we do it?. Chronic Respiratory Disease.

[38] Troosters T, Grosselink R, Decramer M (2000). Short- and long-term effects of outpatient rehabilitation in patients with chronic obstructive pulmonary disease: a randomized trial. Am J Med.

[39] Murphy N, Bell C, Costello RW (2005). Extending a home from hospital care programme for COPD exacerbations to include pulmonary rehabilitation. Respir Med.

[40] Spencer S, Jones PW (2003). Time course of recovery of health status following an infective exacerbation of chronic bronchitis. Thorax.

[41] Troosters T, Gosselink R, Janssens W, Decramer M (2010). Exercise training and pulmonary rehabilitation: new insights and remaining challenges. Eur Respir Rev.

[42] Garcia-Aymerich J, Farrero E, Félez MA, Izquierdo J, Marrades RM, Antó JM (2003). Risk factors of readmission to hospital for a COPD exacerbation: a prospective study. Thorax.

[43] ATS Committee on Proficiency Standards for Clinical Pulmonary Function Laboratories (2002). ATS statement: guidelines for the six-minute walk test. Am J Respir Crit Care Med.

[44] Jones PW (2002). Interpreting thresholds for a clinically significant change in health status in asthma and COPD. Eur Respir J.

[45] Hopkinson NS (2014). What is and what is not post exacerbation pulmonary rehabilitation?. BMJ.

[46] Spruit MA, Rochester CL, Pitta F, Goldstein R, Troosters T, Nici L (2014). A 6-week, home-based, unsupervised exercise training program is not effective in patients with chronic respiratory disease directly following a hospital admission. BMJ.

[47] Wilson Kevin C., Krishnan Jerry A., Sliwinski Pawel, Criner Gerard J., Miravitlles Marc, Hurst John R., Calverley Peter M.A., Albert Richard K., Rigau David, Tonia Thomy, Vestbo Jørgen, Papi Alberto, Rabe Klaus F., Anzueto Antonio, Wedzicha Jadwiga A. (2018). Pulmonary rehabilitation for patients with COPD during and after an exacerbation-related hospitalisation: back to the future?. European Respiratory Journal.

