# Supplementary material for: Lower mortality after early supervised pulmonary rehabilitation following COPD-exacerbations: a systematic review and meta-analysis
Source: BMC Pulm Med. 2018 Sep 15;18:154. doi: 10.1186/s12890-018-0718-1 (PMC6139159; doi:10.1186/s12890-018-0718-1)
Supplement: Supplementary file 1 — Search strategy. The full search strategy from the systematic multidatabase literature search performed in 2013 and 2017. (PDF 214 kb) [file 12890_2018_718_MOESM1_ESM.pdf]

Additional file 1: Search strategy

Overview of searched databases in the first search in 2013:

Database	Interface	Date:
G-I-N International		11.07.2013
NICE (UK)		11.07.2013
National Guideline Clearinghouse (USA)		09.07.2013
Scottish Intercollegiate Guidelines Network (SIGN)		09.07.2013
HTA Database		09.07.2013
The Cochrane Library		09. 07 2013
SBU, Sweden		09.07.2013
Socialstyrelsen, Sweden		11.07.2013
Helsedirektoratet, Norway		10.07.2013
Kunnskapscentret, Norway		10. 07.2013
Pedro		12.07.2013
OT-Seeker		10. 07.2013

Database	Interface	Date:
Medline	Ovid	16.07.2013
EMBASE	Ovid	17.07.2013
CINAHL	Ebsco	16.07.2013
PSYCINFO	OVID	15.07.2013

.

Search strategy in 2013

G-I-N international, 35 records
http://www.g-i-n.net
Search strategy
chronic obstruct* OR copd OR coad, 35 records
chronic pulm*, no new findings
chronic lung, no new findings
chronic airway* no new findings

NICE: 53 records
http://www.nice.org.uk
Search strategy
COPD + guidance, 20 records
COAD + guidance, 1 record, no new findings
obstructive + guidance + respiratory (Condition and diseases), 26 records, 16 new records
chronic obstructive + guidance + respiratory, 18 records , 1 new records
chronic obstructive + guidance, 35 records, 13 new records
chronic obstructive pulmonale + guidance, 2 records, no new findings
chronic obstructive lung + guidance, 26 records, no new findings
chronic obstructive airway + guidance, 18 records, 3 new records

National Guideline Clearing house: 151 records
http://www.guideline.gov
Search strategy:
'chronic obstructive pulmonary or copd or coad or chronic obstructive lung or chronic obstructive airway'

Scottish Intercollegiate Guidelines Network (SIGN): 8 records

Search strategy: Respiratory Medicine

The HTA-database: 74 records
http://www.crd.york.ac.uk/CRDWeb/

Search strategy:

Line	Search	Hits	
1	MeSH DESCRIPTOR Pulmonary Disease, Chronic Obstructive EXPLODE ALL TREES	376	Delete

2	* IN HTA FROM 2002 TO 2013	9073	Delete
3	#1 AND #2	51	Delete
4	(copd) OR (coad) IN HTA FROM 2002 TO 2013	46	Delete
5	(lung) OR (pulmon*) OR (airway*) IN HTA FROM 2002 TO 2013	450	Delete
6	(chronic) AND (obstruct*) IN HTA FROM 2002 TO 2013	80	Delete
7	#5 AND #6	71	Delete
8	#3 OR #4 OR #7	74	Delete

SBU, Sweden, 4 records
http://www.sbu.se/sv/
Search strategy:
kol, 4 records, but only 2 relevant records
kroniskt obstruktiv lungsjukdom 4 records 3 duplicates, 1 new record.
kronisk lunge, no new findings
kronisk obstruktiv, no new findings
lunge, 20 records, 1 new record
Luftvägssjukdom, 1 record, but not relevant

Socialstyrelsen, Sweden, 16 records
http://www.socialstyrelsen.se
Search strategy:
kroniskt obstruktiv lungsjukdom + publikationer, 5, records
kroniskt obstruktiv lungsjukdom + Föreskrifter och allmänna råd, 1 record
KOL + publikationer, 6 records
KOL + Nyheter og konferencer, 4 records

Helsedirektoratet, Norway, 3 records
http://helsedirektoratet.no
Search strategy:
KOLS + Publikasjoner, 1 record
Lungesykdommer, 2 records

Kunnskapssenteret, Norway, 23 records
http://www.kunnskapssenteret.no

Search strategy:
KOLS + *Publikasjoner,* 20 records
KOLS + *Prosjekter*, 0 records
KOLS + *Rehabilitering,* 0 records
Lungesykdom + *Rehabilitering,* 0 records
KOLS + *kvalitetsforbedring*, 1 record, but no new
Lungesykdom + *kvalitetsforbedring*, 0 records
kronisk obstruktiv lungesykdom + Publikasjoner, 10 records, 3 new records
kronisk obstruktiv lungesykdom + Prosjekter, 0 records
lungesykdom + *prosjekter,* 0 records

Pedro, 174 records
http://www.pedro.org.au
Search strategy:
Topic: chronic respiratory disease + Practice guideline, 30 records
Topic: chronic respiratory disease + Systematic review, 144 records

OT-Seeker, 35 records fund
http://www.otseeker.com
Search strategy:
copd OR coad OR chronic obstructive* + Systematic Review 35 records
obstruct* AND lung + systematic review, 7 records, no new findings
obstruct* AND pulmon* + systematic review, 33 records, no new findings
obstruct* AND airway* + systematic review, 8 records, 1 new record, but not relevant.

The Cochrane Library: 275 records fund (80 reviews and 195 other reviews)
http://www.thecochranelibrary.com/
Search strategy:
#1 MeSH descriptor: [Pulmonary Disease, Chronic Obstructive] explode all trees
#2 pulmonary:ti,ab,kw (Word variations have been searched)
#3 lung:ti,ab,kw (Word variations have been searched)
#4 airway:ti,ab,kw (Word variations have been searched)
#5 #2 or #3 or #4
#6 chronic obstructive:ti,ab,kw (Word variations have been searched)
#7 #5 and #6
#8 #1 or #7

Medline (OVID), 275 records fund
Database: Ovid MEDLINE(R) In-Process & Other Non-Indexed Citations and Ovid MEDLINE(R) <1946 to Present>

Search Strategy:
----
1 exp Pulmonary Disease, Chronic Obstructive/ or (copd or coad).mp. (33486)
2 (obstruct* and (pulmon* or lung or airway* or airflow*)).mp. (98524)
3 1 and 2 (31238)
4 1 or 3 (33486)
5 exp guideline/ or guideline*.ti. (62221)
6 4 and 5 (442)
7 limit 6 to ((danish or english or norwegian or swedish) and yr="2002 – Current") (275)

EMBASE (OVID), 1174 records fund
Database: Embase <1974 to 2013 Week 28>

Search Strategy:
----
1 exp *chronic obstructive lung disease/ (40865)
2 practice guideline/ or guideline*.mp. (376951)
3 1 and 2 (2329)
4 limit 3 to ((danish or english or norwegian or swedish) and yr="2002 -Current") (1602)
5 exp chronic obstructive lung disease/ or (copd or coad).mp. (74316)
6 (obstruct* and (pulmon* or lung or airway* or airflow*)).mp. (143296)
7 5 or 6 (148101)
8 practice guideline/ or guideline*.mp. (376951)
9 7 and 8 (6031)
10 limit 9 to ((danish or english or norwegian or swedish) and yr="2013 -Current") (270)
11 4 or 10 (1785)
12 exp rehabilitation/ or (rehab* or occupational therap*).mp. (320155)
13 exp physiotherapy/ or (physical therap* or physiotherap*).mp. (71685)
14 12 or 13 (370613)
15 fitness/ or endurance/ or (physical endurance or physical function).mp. (47768)
16 exp exercise/ or exercise test/ or exp walking/ or (walking or shuttle walking test*).mp. (301013)
17 15 or 16 (328028)
18 exp smoking cessation/ or smoking.mp. (273075)
19 exp nutritional status/ or nutrition/ or exp diet therapy/ or (nutrition* or diet).mp. (731951)
20 body mass/ or (body mass or BMI).mp. (241743)
21 19 or 20 (918999)
22 patient education/ or social support/ (134596)
23 adaptive behavior/ or coping behavior/ or empowerment/ (78274)
24 patient participation/ or exp self care/ (60926)

25 (psychological adaption or coping or empowerment or Self Management or adherence).mp. (166798)
26 22 or 23 or 24 or 25 (359619)
27 14 or 17 or 18 or 21 or 26 (2016728)
28 11 and 27 (959)
29 chronic obstructive lung disease/rh, th and (practice guideline/ or guideline*.mp.) (919)
30 28 or 29 (1529)
31 limit 30 to ((danish or english or norwegian or swedish) and yr="2002 -Current") (1174)

****

CINAHL (EBSCO Host), 404 records fund

Search strategy:

Search ID#	Search Terms	Search Options	Last Run Via	Results
S11	S6 AND S9	Limiters - Published Date from: 20020101-20131231; Language: Danish, English, Norwegian, Swedish<br>Search modes - Boolean/Phrase	Interface - EBSCOhost<br>Search Screen - Advanced Search<br>Database - CINAHL with Full Text	404
S10	S6 AND S9	Search modes - Boolean/Phrase	Interface - EBSCOhost<br>Search Screen - Advanced Search<br>Database - CINAHL with Full Text	464
S9	S7 OR S8	Search modes - Boolean/Phrase	Interface - EBSCOhost<br>Search Screen - Advanced Search<br>Database - CINAHL with Full Text	39,717
S8	TI guideline*	Search modes - Boolean/Phrase	Interface - EBSCOhost<br>Search Screen - Advanced Search<br>Database - CINAHL with Full Text	18,324
S7	(MH "Practice Guidelines")	Search modes - Boolean/Phrase	Interface - EBSCOhost<br>Search Screen - Advanced Search<br>Database - CINAHL with Full Text	30,146
S6	S1 OR S2 OR S5	Search modes - Boolean/Phrase	Interface - EBSCOhost<br>Search Screen - Advanced Search<br>Database - CINAHL with Full Text	16,134
S5	S3 AND S4	Search modes - Boolean/Phrase	Interface - EBSCOhost<br>Search Screen -	15,714

			Advanced Search<br>Database - CINAHL with Full Text	
S4	obstruct*	Search modes - Boolean/Phrase	Interface - EBSCOhost<br>Search Screen - Advanced Search<br>Database - CINAHL with Full Text	23,334
S3	pulmon* OR lung OR airway* OR airflow*	Search modes - Boolean/Phrase	Interface - EBSCOhost<br>Search Screen - Advanced Search<br>Database - CINAHL with Full Text	75,205
S2	copd OR coad	Search modes - Boolean/Phrase	Interface - EBSCOhost<br>Search Screen - Advanced Search<br>Database - CINAHL with Full Text	5,355
S1	(MH "Pulmonary Disease, Chronic Obstructive+")	Search modes - Boolean/Phrase	Interface - EBSCOhost<br>Search Screen - Advanced Search<br>Database - CINAHL with Full Text	6,618

PsycINFO, 55 records fund
Database: PsycINFO <1806 to July Week 2 2013>

Search Strategy:
----
1 exp Chronic Obstructive Pulmonary Disease/ (681)
2 (copd or coad).mp. (849)
3 (obstruct* and (pulmon* or lung or airway*)).mp. (2177)
4 1 or 2 or 3 (2399)
5 treatment guidelines/ (3872)
6 guideline*.mp. (40056)
7 5 or 6 (40056)
8 4 and 7 (61)
9 limit 8 to ((danish or english or norwegian or swedish) and yr="2002 – Current") (55)
****

Second search in 2013:

Systematic Reviews, Meta-analysis and guidelines
Medline og Embase, 134 records fund, after duplicate search: 108 records.

Search conducted: 11.11.2013

Medline, 46 records fund.
Database: Ovid MEDLINE(R) In-Process & Other Non-Indexed Citations and Ovid MEDLINE(R) <1946 to Present>

Search Strategy:
----
1 exp Pulmonary Disease, Chronic Obstructive/ or (copd or coad).mp. (35070)
2 Weight Lifting/ (4003)
3 exp Resistance Training/ (3134)
4 motion therapy, continuous passive/ or muscle stretching exercises/ (1465)
5 static exercise.ti,ab. (423)
6 muscle training.ti,ab. (1484)
7 isometric exercise*.ti,ab. (1611)
8 isokinetic exercise*.ti,ab. (234)
9 (anaerobic exercise* or aerobic exercise*).ti,ab. (5505)
10 Strength training.ti,ab. (2930)
11 workout*.ti,ab. (656)
12 body building.ti,ab. (170)
13 exp Muscle Strength/ (17870)
14 2 or 3 or 4 or 5 or 6 or 7 or 8 or 9 or 10 or 11 or 12 or 13 (34948)
15 1 and 14 (468)
16 meta-analysis/ (51581)
17 exp guideline/ (25320)
18 (systematic review or meta analysis).ti,ab. (86887)
19 exp Evidence-Based Medicine/ (55611)
20 16 or 17 or 18 or 19 (177722)
21 15 and 20 (41)
22 limit 15 to (guideline or meta analysis or practice guideline or systematic reviews) (44)
23 21 or 22 (46)

****

Embase, 88 records fund
Database: Embase <1980 to 2013 Week 45>

Search Strategy:
----
1 exp Pulmonary Disease, Chronic Obstructive/ or (copd or coad).mp. (75361)
2 exp resistance training/ (4683)
3 exp weight lifting/ (3744)
4 arm exercise/ or isokinetic exercise/ or isometric exercise/ or muscle training/ or static exercise/ (10072)
5 workout*.ti,ab. (845)
6 (anaerobic exercise* or aerobic exercise*).ti,ab. (6705)
7 Strength training.ti,ab. (3313)
8 body building.ti,ab. (280)
9 muscle training.ti,ab. (1828)
10 muscle stretching.ti,ab. (277)
11 exp muscle strength/ (30912)
12 2 or 3 or 4 or 5 or 6 or 7 or 8 or 9 or 10 or 11 (52574)
13 1 and 12 (1266)
14 evidence based medicine/ or consensus development/ or meta analysis/ or "meta analysis (topic)"/ or outcomes
research/ or "systematic review"/ or "systematic review (topic)"/ (271326)
15 13 and 14 (103)
16 limit 13 to (evidence based medicine or concensus development or meta analysis or outcomes research or
"systematic review") (96)
17 15 or 16 (103)
18 limit 17 to ((danish or english or norwegian or swedish) and yr="2002 -Current") (87)
19 (systematic review or meta analysis).ti. (63018)
20 14 or 19 (284674)
21 13 and 20 (106)
22 16 or 21 (106)
23 limit 22 to ((danish or english or norwegian or swedish) and yr="2002 -Current") (88)

****

Randomized Controlled Trials in 2013

Search in MEDLINE and EMBASE . Search conducted: 27.11.2013. We found 1254 records, after duplicate search we found 750 records.

Medline, 649 records found.

Database: Ovid MEDLINE(R) In-Process & Other Non-Indexed Citations and Ovid MEDLINE(R) <1946 to Present>

Search Strategy:

----

1 Weight Lifting/ (4008)
2 exp Resistance Training/ (3160)
3 motion therapy, continuous passive/ or muscle stretching exercises/ (1472)
4 static exercise.ti,ab. (424)
5 muscle training.ti,ab. (1488)
6 isometric exercise*.ti,ab. (1613)
7 isokinetic exercise*.ti,ab. (235)
8 (anaerobic exercise* or aerobic exercise*).ti,ab. (5532)
9 Strength training.ti,ab. (2947)
10 workout*.ti,ab. (663)
11 body building.ti,ab. (170)
12 exp Muscle Strength/ (17961)
13 exp Exercise Movement Techniques/ (5321)
14 exp *Exercise Test/ (14746)
15 Exercise Tolerance/ (8505)
16 *Physical Endurance/ (8937)
17 (exercise adj (testing or tolerance or capacity)).ti,ab. (20868)
18 ((stress or treadmill or step) adj testing).ti,ab. (4933)
19 (shuttle adj2 walk*).ti,ab. (389)
20 1 or 2 or 3 or 4 or 5 or 6 or 7 or 8 or 9 or 10 or 11 or 12 or 13 or 14 or 15 or 16 or 17 or 18 or 19 (83767)
21 exp Pulmonary Disease, Chronic Obstructive/ (24710)
22 (chronic obstructive pulmonary disease or coad or copd).ti,ab. (38238)
23 (chronic obstructive lung disease or lung disease chronic obstructive).ti,ab. (3034)
24 21 or 22 or 23 (44777)
25 20 and 24 (3311)
26 randomized controlled trial/ or ((randomized or randomised) adj2 trial).ti,ab. or rct.ti,ab. (425466)
27 25 and 26 (687)
28 limit 27 to (danish or english or norwegian or swedish) (649)

Embase , 605 records found

Database: Embase <1974 to 2013 November 26>

Search Strategy:

----

1 exp resistance training/ (4781)
2 exp weight lifting/ (3821)
3 arm exercise/ or isokinetic exercise/ or isometric exercise/ or muscle training/ or static exercise/ (10353)
4 workout*.ti,ab. (855)
5 (anaerobic exercise* or aerobic exercise*).ti,ab. (6756)
6 Strength training.ti,ab. (3357)
7 body building.ti,ab. (286)
8 muscle training.ti,ab. (1864)
9 muscle stretching.ti,ab. (289)
10 muscle training/ (4714)
11 exp *kinesiotherapy/ (22914)
12 exp *exercise/ or exp muscle exercise/ (104828)
13 (exercise adj (testing or tolerance or capacity)).ti,ab. (26741)
14 ((stress or treadmill or step) adj testing).ti,ab. (6442)
15 (shuttle adj2 walk*).ti,ab. (536)
16 exercise tolerance/ (10823)
17 1 or 2 or 3 or 4 or 5 or 6 or 7 or 8 or 9 or 10 or 11 or 12 or 13 or 14 or 15 or 16 (162372)
18 exp chronic obstructive lung disease/ (70846)
19 (chronic obstructive pulmonary disease or coad or copd).ti,ab. (49748)
20 (chronic obstructive lung disease or lung disease chronic obstructive).ti,ab. (4080)
21 18 or 19 or 20 (81194)
22 17 and 21 (5021)
23 randomized controlled trial/ or ((randomized or randomised) adj2 trial).ti,ab. or rct.ti,ab. (414459)
24 22 and 23 (655)
25 limit 24 to (danish or english or norwegian or swedish) (605)

PEDRO, 89 records found

We found 89 records, after duplicate search: 18 new records.

Search conducted: 11.12.2013

Search strategy:

Therapy: strength training AND topic: chronic respiratory disease AND Method: clinical trial

From year 2002 -

Search strategy in 2017

Overview of searched databases in the first search for guidelines and systematic reviews in 2017:

Database	Interface	Fund	Date
G-I-N International	Internet	(49) 9	26.06.2017
NICE (UK)	-	(54) 4	26.06.2017
National Guideline Clearinghouse (USA)	-	(27) 8	26.06.2017
Scottish Intercollegiate Guidelines Network (SIGN)	-	None	26.06.2017
HTA Databasen (CRD database)	-	31	26.06.2017
SBU, Sweden	-	4	26.06.2017
Socialstyrelsen, Sweden	-	4	26.06.2017
Helsedirektoratet, Norway	-	None	26.06.2017
Kunnskapssenteret, Norway	-	None	26.06.2017
The Cochrane library	-	70	16.09.2017
Medline	OVID	218	07.09.2017
Embase	OVID	256	07.09.2017

Search strategy in 2017

G-I-N international: 49 records, 9 relevant
http://www.g-i-n.net
Search strategy:
COPD or chronic obstructive pulmonary disease

NICE: 54 records, 4 relevant
http://www.nice.org.uk
Search strategy:
COPD or chronic obstructive pulmonary disease

National Guideline Clearinghouse: 27 records, 8 relevant
http://www.guideline.gov
Search strategy:
COPD or chronic obstructive pulmonary disease

CRD/HTA: 31 records
http://www.crd.york.ac.uk/CRDWeb/

Search strategy:

Line	Search	Hits
1	(COPD OR chronic obstructive pulmonary disease*) IN HTA FROM 2013 TO 2017	31

SBU, Sweden: 4 records
http://www.sbu.se/sv/
Search strategy:
kroniskt obstruktiv lungsjukdom
KOL

Socialstyrelsen, Sweden: 4 records
http://www.socialstyrelsen.se
Search strategy:
kroniskt obstruktiv lungsjukdom
KOL

Medline (OVID): 218 records fund
Database(s): Ovid MEDLINE(R) Epub Ahead of Print, In-Process & Other Non-Indexed Citations, Ovid MEDLINE(R) Daily, Ovid MEDLINE and Versions(R)

Search strategy:

#	Searches	Results
1	exp Pulmonary Disease, Chronic Obstructive/	48705
2	(chronic obstructive lung adj (disease or disorder*)).ti,kw.	1171
3	(chronic obstructive pulmonary adj (disease or disorder*)).ti,kw.	16086

4	(chronic obstructive airway* adj (disease or disorder*)).ti,kw.	249
5	(copd or coad or kol).ti,kw.	17351
6	or/1-5	59213
7	(Guideline* or practice guideline* or clinical guideline* or guidance or recommendation*).ti,kw.	114688
8	6 and 7	636
9	limit 6 to (guideline or practice guideline)	164
10	8 or 9	682
11	limit 10 to (yr="2013-2017" and (danish or english or french or german or norwegian or swedish))	218

Embase (OVID): 256 records fund
Database(s): **Embase** 1974 to 2017 September 06

Search strategy:

#	**Searches**	**Results**
1	exp chronic obstructive lung disease/	104177
2	(chronic obstructive lung adj (disease or disorder*)).ti,kw. and (2015* or 2016* or 2017*).em.	1771
3	(chronic obstructive pulmonary adj (disease or disorder*)).ti,kw. and (2015* or 2016* or 2017*).em.	25991
4	(chronic obstructive airway* adj (disease or disorder*)).ti,kw. and (2015* or 2016* or 2017*).em.	378
5	(copd or coad or kol).ti,kw. and (2015* or 2016* or 2017*).em.	31486
6	or/1-5	111380
7	*practice guideline/	53580
8	6 and 7	619
9	limit 8 to (yr="2013-2017" and (danish or english or french or german or norwegian or swedish))	256

Cochrane Reviews: 70 records fund

ID	Search	Hits
#1	MeSH descriptor: [Pulmonary Disease, Chronic Obstructive] explode all trees	3428
#2	(chronic obstructive lung near (disease or disorder*)):ti,kw	3737
#3	(chronic obstructive pulmonary near (disease or disorder*)):ti,kw	5853
#4	(chronic obstructive airway* near/2 (disease or disorder*)):ti,kw	144
#5	(copd or coad or kol):ti,kw	7311
#6	#1 or #2 or #3 or #4 or #5	12656
#7	#6 Publication Year from 2013 to 2017, in Cochrane Reviews (Reviews and Protocols)	70

Second search for primary studies (Randomized Controlled Trials) in 2017

Overview over searched databases in the second search in 2017:

Database	Interface	Fund	Date
Medline	OVID	787	06.10.2017
Embase	OVID	1083	06.10.2017
Cinahl	EbsCo	102	06.10.2017
Pedro	Internet	121	06.10.2017
After duplicate sorting		1187	

Medline
Database(s): Ovid MEDLINE(R) Epub Ahead of Print, In-Process & Other Non-Indexed Citations, Ovid MEDLINE(R) Daily, Ovid MEDLINE and Versions(R)

Search Strategy:

#	Searches	Results
1	exp Pulmonary Disease, Chronic Obstructive/	49983
2	(chronic obstructive pulmonary disease or coad or copd).ti,ab,kw.	54594
3	(chronic obstructive lung disease or lung disease chronic obstructive).ti,ab,kw.	3925
4	or/1-3	77102
5	exp Rehabilitation/ or exp “Delivery of Health Care, Integrated”/	292185
6	4 and 5	3156
7	((rehabilitation or integrated or Multi-modal* or multimodal* or multi-component or multicomponent or multidisciplinary or multi-disciplinary or interdisciplinary or inter-disciplinary or complimentary or different or multiple or addition*) adj3 (program* or intervention* or treatment* or therap* or training or exercise* or care or modalities)).ti,ab,kw.	294224
8	4 and 7	2495
9	"Pulmonary Disease, Chronic Obstructive"/rh	2023
10	(Pulmonary adj1 Rehabilitation).ti,ab,kw.	2901

11	((chronic obstructive pulmonary disease or coad or copd or chronic obstructive lung disease) and (rehabilitation or program*)).ti,ab,kw.	5051
12	or/6,8-11	8839
13	limit 12 to (randomized controlled trial or controlled clinical trial)	1263
14	(((random* or control?ed or crossover or cross-over or blind* or mask*) adj3 (trial*1 or study or studies or analy*)) or rct).ti,ab,kw.	598707
15	(placebo* or single-blind* or double-blind* or triple-blind*).ti,ab,kw.	272653
16	((single or double or triple) adj2 (blind* or mask*)).ti,ab,kw.	168315
17	(patient* adj3 (random* or blind* or mask*)).ti,ab,kw.	104006
18	or/14-17	729035
19	12 and 18	1505
20	13 or 19	1905
21	limit 20 to (yr="2013-2017" and (english or danish or german or norwegian or swedish))	787

Embase
Database(s): Embase 1974 to 2017 October 03

Search Strategy:

#	**Searches**	**Results**
1	exp Chronic Obstructive Pulmonary Disease/	104825
2	(chronic obstructive pulmonary disease or coad or copd).ti,ab,kw.	88899
3	(chronic obstructive lung disease or lung disease chronic obstructive).ti,ab,kw.	5405
4	or/1-3	127951
5	((rehabilitation or integrated or Multi-modal* or multimodal* or multi-component or multicomponent or multidisciplinary or multi-disciplinary or interdisciplinary or inter-disciplinary or complimentary or different or multiple or addition*) adj3 (program* or intervention* or treatment* or therap* or training or exercise* or care or modalities)).ti,ab,kw.	345440
6	4 and 5	4513
7	Chronic Obstructive Pulmonary Disease/rh	950
8	(Pulmonary adj1 Rehabilitation).ti,ab,kw.	5212

9	((copd or chronic obstructive pulmonary disease) and (rehabilitation or program*)).ti,ab,kw.	8893
10	6 or 7 or 8 or 9	12569
11	limit 10 to (randomized controlled trial or controlled clinical trial)	1297
12	(((random* or control?ed or crossover or cross-over or blind* or mask*) adj3 (trial*1 or study or studies or analy*)) or rct).ti,ab,kw.	748848
13	(placebo* or single-blind* or double-blind* or triple-blind*).ti,ab,kw.	341609
14	((single or double or triple) adj2 (blind* or mask*)).ti,ab,kw.	207721
15	(patient* adj3 (random* or blind* or mask*)).ti,ab,kw.	143336
16	or/12-15	919107
17	10 and 16	1917
18	11 or 17	2268
19	limit 18 to (yr="2013-2017" and (english or danish or german or norwegian or swedish))	1063

Cinahl

Search strategy:

#	Query	Limiters/Expanders	Results
S11	S9 AND S10	Limiters - Published Date: 20130101-20171031; Language: Danish, English, German, Norwegian, Swedish	102
S10	PT Randomized Controlled Trial		31,565
S9	S6 OR S7 OR S8		10,647
S8	TX ((chronic obstructive pulmonary disease or coad or copd or chronic obstructive lung disease) and (rehabilitation or program*))		10,145
S7	TI (Pulmonary N1 Rehabilitation)		716
S6	S4 AND S5		994
S5	TX ((rehabilitation or integrated or Multi-modal* or multimodal* or multi-component or multicomponent or multidisciplinary or multi-disciplinary or interdisciplinary or inter-		149,149

	disciplinary or complimentary or different or multiple or addition*) N3 (program* or intervention* or treatment* or therap* or training or exercise* or care or modalities))		
S4	S1 OR S2 OR S3		10,515
S3	TI (chronic obstructive lung disease or lung disease chronic obstructive)		77
S2	TI (chronic obstructive pulmonary disease or coad or copd)		7,850
S1	(MH "Pulmonary Disease, Chronic Obstructive+")		9,486

PEDRO: 121 records fund
Abstract & Title: COPD Rehabilitation or COPD Program*
Method: Clinical trial
Published Since: 2013
