# Supplementary material for: Lower mortality after early supervised pulmonary rehabilitation following COPD-exacerbations: a systematic review and meta-analysis
Source: BMC Pulm Med. 2018 Sep 15;18:154. doi: 10.1186/s12890-018-0718-1 (PMC6139159; doi:10.1186/s12890-018-0718-1)
Supplement: Supplementary file 2 — AGREE II. A critical group appraisal of: Pulmonary rehabilitation for patients with chronic pulmonary disease (COPD): an evidence-based analysis using the AGREE II Instrument. (PDF 54 kb) [file 12890_2018_718_MOESM2_ESM.pdf]

A critical group appraisal of: Pulmonary rehabilitation for patients with chronic pulmonary disease (COPD): an evidence-based analysis using the AGREE II Instrument

Created with the AGREE II Online Guideline Appraisal Tool.

No endorsement of the content of this document by the AGREE Research Trust should be implied.

Co-ordinator: Ulrik Winning Iepsen

Date: 25 October 2013

Email: ulrik_winning@hotmail.com

URL of this appraisal: http://www.agreetrust.org/group-appraisal/210

Guideline URL:
www.hqontario.ca/en/mas/tech/pdfs/2012/rev_Pulmomary_Rehab_March.pdf
