# Supplementary material for: Lower mortality after early supervised pulmonary rehabilitation following COPD-exacerbations: a systematic review and meta-analysis
Source: BMC Pulm Med. 2018 Sep 15;18:154. doi: 10.1186/s12890-018-0718-1 (PMC6139159; doi:10.1186/s12890-018-0718-1)
Supplement: Supplementary file 3 — AMSTAR (A Measurement Tool to Asses Systematic Reviews). An assessment of the methodological quality of the included systematic reviews. (PDF 10 kb) [file 12890_2018_718_MOESM3_ESM.pdf]

Additional file 3. AMSTAR (A Measurement Tool to Asses Systematic Reviews) assessment of the included systematic reviews.

Observer	Citation	Was meta-analysis performed for the relevant outcomes?	1. Was an 'a priori' design provided?	2. Was there duplicate study selection and data extraction?	3. Was a comprehensive literature search performed?	4. Was the status of publication (i.e. grey literature) used as an inclusion criterion?	5. Was a list of studies (included and excluded) provided?	6. Were the characteristics of the included studies provided?	7. Was the scientific quality of the included studies assessed and documented?	8. Was the scientific quality of the included studies used appropriately in formulating conclusions?	9. Were the methods used to combine the findings of studies appropriately in formulating conclusions?	10. Was the likelihood of publication bias assessed?	11. Was the conflict of interest included?	**Total score**
Consensus	Puhan 2011	Yes	Yes	Yes	Yes	Yes	Yes	Yes	Yes	Yes	Yes	No	Yes	**10**
Consensus	Puhan 2016	Yes	Yes	Yes	Yes	Yes	Yes	Yes	Yes	Yes	Yes	No	Yes	**10**
