# Supplementary material for: Lower mortality after early supervised pulmonary rehabilitation following COPD-exacerbations: a systematic review and meta-analysis
Source: BMC Pulm Med. 2018 Sep 15;18:154. doi: 10.1186/s12890-018-0718-1 (PMC6139159; doi:10.1186/s12890-018-0718-1)
Supplement: Supplementary file 4 — Assessment of the included studies. Characteristics and risk of bias assessment of the included studies. (PDF 329 kb) [file 12890_2018_718_MOESM4_ESM.pdf]

Additional file 4. Characteristics of included studies

Behnke 2000

Methods	Randomized parallel group trial
Participants	26 COPD patients (mean age 67 years, 77% males, mean FEV_1=36% predicted) after inpatient treatment for acute exacerbation
Interventions	Rehabilitation: Within 4-7 days after admission, inpatient pulmonary rehabilitation with endurance exercise (5 walking sessions/day for 10 days), followed by six months of supervised home-based endurance exercise (3 walking sessions/day for 6 months). Completion rate of pulmonary rehabilitation of 65.2% (15 out of 23 patients)<br>Usual care: Standard inpatient care without exercise and standard community care with respirologist. Follow-up: 76 weeks
Outcomes	Lung function, health-related quality of life (HRQoL), walking test (6MWT), breathlessness, readmissions
Notes	

<u>Risk of bias table:</u>

Bias	Author’s judgement	Support for judgement
Random sequence generation (selection bias)	Unclear risk	See Giacomini, M., D. DeJean, and D. Simeonov. "PULMONARY REHABILITATION FOR PATIENTS WITH CHRONIC PULMONARY DISEASE (COPD): AN EVIDENCE BASED ANALYSIS." *Ont Health Technol Assess Ser* 12 (2012): 1-47.
Allocation concealment (selection bias)	Low risk	See Giacomini, M., D. DeJean, and D. Simeonov. "PULMONARY REHABILITATION FOR PATIENTS WITH CHRONIC PULMONARY DISEASE (COPD): AN EVIDENCE BASED ANALYSIS." *Ont Health Technol Assess Ser* 12 (2012): 1-47.
Blinding of participants and personnel (performance bias) Hospital admission	Unclear risk	See Giacomini, M., D. DeJean, and D. Simeonov. "PULMONARY REHABILITATION FOR PATIENTS WITH CHRONIC PULMONARY DISEASE (COPD): AN EVIDENCE BASED ANALYSIS." *Ont Health Technol Assess Ser* 12 (2012): 1-47.
Blinding of participants	High risk	See Giacomini, M., D. DeJean, and D. Simeonov. "PULMONARY REHABILITATION FOR

and personnel (performance bias) HRQoL		PATIENTS WITH CHRONIC PULMONARY DISEASE (COPD): AN EVIDENCE BASED ANALYSIS." *Ont Health Technol Assess Ser* 12 (2012): 1-47.
Blinding of participants and personnel (performance bias) Mortality	Low risk	See Giacomini, M., D. DeJean, and D. Simeonov. "PULMONARY REHABILITATION FOR PATIENTS WITH CHRONIC PULMONARY DISEASE (COPD): AN EVIDENCE BASED ANALYSIS." *Ont Health Technol Assess Ser* 12 (2012): 1-47.
Blinding of participants and personnel (performance bias) Walk test	Unclear risk	See Giacomini, M., D. DeJean, and D. Simeonov. "PULMONARY REHABILITATION FOR PATIENTS WITH CHRONIC PULMONARY DISEASE (COPD): AN EVIDENCE BASED ANALYSIS." *Ont Health Technol Assess Ser* 12 (2012): 1-47.
Blinding of outcome assessment (detection bias)	Low risk	See Giacomini, M., D. DeJean, and D. Simeonov. "PULMONARY REHABILITATION FOR PATIENTS WITH CHRONIC PULMONARY DISEASE (COPD): AN EVIDENCE BASED ANALYSIS." *Ont Health Technol Assess Ser* 12 (2012): 1-47.
Incomplete outcome data (attrition bias)	High risk	See Giacomini, M., D. DeJean, and D. Simeonov. "PULMONARY REHABILITATION FOR PATIENTS WITH CHRONIC PULMONARY DISEASE (COPD): AN EVIDENCE BASED ANALYSIS." *Ont Health Technol Assess Ser* 12 (2012): 1-47.
Selective reporting (reporting bias)	Unclear risk	See Giacomini, M., D. DeJean, and D. Simeonov. "PULMONARY REHABILITATION FOR PATIENTS WITH CHRONIC PULMONARY DISEASE (COPD): AN EVIDENCE BASED ANALYSIS." *Ont Health Technol Assess Ser* 12 (2012): 1-47.
Other bias	Unclear risk	See Giacomini, M., D. DeJean, and D. Simeonov. "PULMONARY REHABILITATION FOR PATIENTS WITH CHRONIC PULMONARY DISEASE (COPD): AN EVIDENCE BASED ANALYSIS." *Ont Health Technol Assess Ser* 12 (2012): 1-47.

Daabis 2017

Methods	Study design: Randomized controlled trial<br>Study grouping: Parallel group
Participants	Baseline Characteristics<br>Intervention 1<br>• *COPD severity (GOLD/MRC score)*: 2.62 (0.76) MRC, 53.2(9.5) FEV_1%<br>• *Age (range)*: 61 (8) years

	Intervention 2<br>• *COPD severity (GOLD/MRC score)*: 2.58 (0.69) MRC, 56.4(8.3) FEV_1%<br>• *Age (range)*: 58 (7) years<br>Control<br>• *COPD severity (GOLD/MRC score)*: 2.53 (0.89) MRC; 54.6(7.1) FEV_1%<br>• *Age (range)*: 60 (8) years<br><br>Included criteria: Patients admitted to chest dis-eases department, Alexandria Main University Hospital witha primary diagnosis of acute exacerbation of COPD.<br><br>Excluded criteria: Exclusion criteria: 1) Hypoxemic patients at rest or exercise, 2) Comorbidity that could limit exercise training like cardiovascular, musculoskeletal or neuromuscular diseases, 3) Patients who attended a pulmonary rehabilitation program in the preceding year.<br><br>Pretreatment: No significant differences were found between groups in terms of age, BMI, airflow obstruction, or arterial Blood gases
Interventions	Intervention Characteristics<br>Intervention 1<br>• *Description*: Endurance training<br>• *Duration (weeks)*: 8 weeks<br>• *Longest follow up (after end of treatment)*: After end of treatment<br><br>Intervention 2<br>• *Description*: Combined training (CT) (endurance + strength training)<br>• *Duration (weeks)*: 8 weeks<br>• *Longest follow up (after end of treatment)*: After end treatment<br><br>Control<br>• *Description*: Medical treatment<br>• *Duration (weeks)*: 8 weeks<br>• *Longest follow up (after end of treatment)*: After end of treatment
Outcomes	*Quality of life, SD*

	• Outcome type: Continuous Outcome<br><br>*6 min Walk test, SD*<br>• Outcome type: Continuous Outcome
Notes	Country: Egypt<br>Authors name: Rasha Daabis<br>Institution: Dept. of Chest Diseases, Faculty of Medicine, Alexandria University, Alexandria, Egypt<br>Email: rgdaabis@yahoo.com, rgdaabis@gmail.com<br>Address: Department of Chest Diseases, Faculty of Medicine, Alexandria University, Alazarita, Alkhartoom Square, Egypt<br>Outcomes<br>• Quality of life: SGRQ, St. Georges Respiratory Questionnaire<br>• Walk test: 6-min test

Risk of bias table:

Bias	Author's judgement	Support for judgement
Random sequence generation (selection bias)	Unclear risk	Patients were allocated randomly to groups. It is unknown how this was done
Allocation concealment (selection bias)	Unclear risk	Nothing mentioned
Blinding of participants and personnel (performance bias) Hospital admission	Unclear risk	Nothing mentioned
Blinding of participants and personnel (performance bias) HRQoL	Unclear risk	Nothing mentioned
Blinding of participants	Unclear risk	Nothing mentioned

and personnel<br>(performance bias)<br>Mortality		
Blinding of participants and personnel (performance bias)<br>Walk test	Unclear risk	Nothing mentioned
Blinding of outcome assessment<br>(detection bias)	Unclear risk	Nothing mentioned
Incomplete outcome data<br>(attrition bias)	Unclear risk	45 patients were enrolled in the study. Only 15 per group was assessed. Nothing mentioned on dropouts.
Selective reporting<br>(reporting bias)	Unclear risk	No other apparent source of bias
Other bias	Unclear risk	No other apparent source of bias

Deepak 2014

Methods	Study design: Randomized controlled trial<br>Study grouping: Parallel group
Participants	Baseline Characteristics<br>Intervention<br>• *COPD severity (GOLD/MRC score)*: 53.3±18.4 (mean FEV_1%)<br>• *Male (%)*: 28 males<br>• *Age (range)*: 58.4 (6.8) age, years<br><br>Control<br>• *COPD severity (GOLD/MRC score)*: 46.7±14.8 (mean FEV_1%)<br>• *Male (%)*: 28 males

	• *Age (range)*: 59.4 (6.7) age, years<br><br>Included criteria: Consecutive patients who were admitted with an AECOPD and were discharged from the hospital Who fulfilled the study criteria were included in the study. Unknown what the inclusion criteria were<br><br>Excluded criteria: Severely ill patients who were unable to walk, or patients with unstable cardiovascular disease (unstable angina or recent acute myocardial infarction), had cognitive impairment, disabling arthritis, and Severe neurological disease was excluded from the study<br><br>Pretreatment: The mean FEV_1% in the case and control group was53.3±18.4 and 46.7±14.8, respectively. The mMRC Breathlessness Scale in the two groups during the initial assessment was found to be similar
Interventions	Intervention Characteristics<br>Intervention<br>• *Description*: Standard treatment plus 12-week post-excerbation pulmonary rehabilitation programme<br>• *Duration (weeks)*: 12 weeks<br>• *Longest follow up (after end of treatment)*: After end of treatment<br><br>Control<br>• *Description*: Conventional treatment without pulmonary rehabilitation<br>• *Duration (weeks)*: 12 weeks<br>• *Longest follow up (after end of treatment)*: After end of treatment
Outcomes	*Quality of life, SD*<br>• Outcome type: Continuous outcome<br><br>*Walk test, SD*<br>• Outcome type: Continuous outcome
Notes	Country: India<br>Setting: in the department of Pulmonary Medicine at Government Medical College Hospital, Chandigarh<br>Authors name: Deepak TH<br>Institution: Department of Pulmonary Medicine, Government Medical College and Hospital<br>Email: prmohapatra@hotmail.com<br>Address: Department of Pulmonary Medicine, All India Institute of Medical Sciences, Bhubaneswar-751 019 (Odisha),

	India.

Risk of bias table:

Bias	Author's judgement	Support for judgement
Random sequence generation (selection bias)	Low risk	Randomisation was done by block randomisation technique.
Allocation concealment (selection bias)	Unclear risk	Nothing mentioned
Blinding of participants and personnel (performance bias) Hospital admission	Unclear risk	Nothing mentioned
Blinding of participants and personnel (performance bias) HRQoL	Unclear risk	Nothing mentioned
Blinding of participants and personnel (performance bias) Mortality	Unclear risk	Nothing mentioned
Blinding of participants and personnel (performance bias) Walk test	Unclear risk	Nothing mentioned
Blinding of outcome assessment (detection bias)	Unclear risk	Nothing mentioned
Incomplete outcome data (attrition bias)	Unclear risk	There were 60 patients enrolled, yet only 28 participants were included in the analysis. There is nothing stated on dropouts.
Selective reporting	Unclear risk	No other apparent source of bias

(reporting bias)		
Other bias	Unclear risk	The inclusion criteria are not stated.

Eaton 2009

Methods	RCT
Participants	N = 97, Rehabilitation: N = 47, control: N = 50
Interventions	Rehabilitation: The patient started inpatient program as soon as medically appropriate as determined by the attending medical team. Inpatient program: Supervised walking and upper-lower limb strengthening exercise at least 30min/day until discharge, followed by outpatient program: supervised exercise for 8 weeks (1 h session, twice weekly) and patient education (coping with dyspnea, the importance of a regular daily home exercise program, management of activities of daily living, drugs, vaccines, airway clearance techniques, nutritional advice, self-management and action plans for exacerbations, stress and panic management, relaxation techniques, mood disturbance, adapting to a chronic illness and end-of-life care). Only 19 (40%) patients assigned to early rehabilitation satisfied the a priori definition of adherence (attendance at 75% of rehabilitation sessions)<br>Follow-up: 12 weeks<br><br>Usual care: Standardized care in accordance with the ATS/ERS COPD guidelines and standardized advice on exercise and maintaining daily activities, but not further specified.<br>Follow-up:12 weeks
Outcomes	BMI, airflow obstruction, breathlessness, walking test (6MWT), HRQoL, readmissions, days in hospital
Notes	Follow-up: 3 months from baseline

<u>Risk of bias table:</u>

Bias	Author’s judgement	Support for judgement
Random sequence generation (selection bias)	Low risk	See Giacomini, M., D. DeJean, and D. Simeonov. "PULMONARY REHABILITATION FOR PATIENTS WITH CHRONIC PULMONARY DISEASE (COPD): AN EVIDENCE BASED ANALYSIS." *Ont Health Technol Assess Ser* 12 (2012): 1-47.
Allocation concealment (selection bias)	Unclear risk	See Giacomini, M., D. DeJean, and D. Simeonov. "PULMONARY REHABILITATION FOR PATIENTS WITH CHRONIC PULMONARY DISEASE (COPD): AN EVIDENCE BASED

		ANALYSIS." *Ont Health Technol Assess Ser* 12 (2012): 1-47.
Blinding of participants and personnel (performance bias) Hospital admission	High risk	See Giacomini, M., D. DeJean, and D. Simeonov. "PULMONARY REHABILITATION FOR PATIENTS WITH CHRONIC PULMONARY DISEASE (COPD): AN EVIDENCE BASED ANALYSIS." *Ont Health Technol Assess Ser* 12 (2012): 1-47.
Blinding of participants and personnel (performance bias) HRQoL	High risk	See Giacomini, M., D. DeJean, and D. Simeonov. "PULMONARY REHABILITATION FOR PATIENTS WITH CHRONIC PULMONARY DISEASE (COPD): AN EVIDENCE BASED ANALYSIS." *Ont Health Technol Assess Ser* 12 (2012): 1-47.
Blinding of participants and personnel (performance bias) Mortality	Low risk	See Giacomini, M., D. DeJean, and D. Simeonov. "PULMONARY REHABILITATION FOR PATIENTS WITH CHRONIC PULMONARY DISEASE (COPD): AN EVIDENCE BASED ANALYSIS." *Ont Health Technol Assess Ser* 12 (2012): 1-47.
Blinding of participants and personnel (performance bias) Walk test	High risk	See Giacomini, M., D. DeJean, and D. Simeonov. "PULMONARY REHABILITATION FOR PATIENTS WITH CHRONIC PULMONARY DISEASE (COPD): AN EVIDENCE BASED ANALYSIS." *Ont Health Technol Assess Ser* 12 (2012): 1-47.
Blinding of outcome assessment (detection bias)	High risk	See Giacomini, M., D. DeJean, and D. Simeonov. "PULMONARY REHABILITATION FOR PATIENTS WITH CHRONIC PULMONARY DISEASE (COPD): AN EVIDENCE BASED ANALYSIS." *Ont Health Technol Assess Ser* 12 (2012): 1-47.
Incomplete outcome data (attrition bias)	Low risk	See Giacomini, M., D. DeJean, and D. Simeonov. "PULMONARY REHABILITATION FOR PATIENTS WITH CHRONIC PULMONARY DISEASE (COPD): AN EVIDENCE BASED ANALYSIS." *Ont Health Technol Assess Ser* 12 (2012): 1-47.
Selective reporting (reporting bias)	Low risk	See Giacomini, M., D. DeJean, and D. Simeonov. "PULMONARY REHABILITATION FOR PATIENTS WITH CHRONIC PULMONARY DISEASE (COPD): AN EVIDENCE BASED ANALYSIS." *Ont Health Technol Assess Ser* 12 (2012): 1-47.
Other bias	Unclear risk	See Giacomini, M., D. DeJean, and D. Simeonov. "PULMONARY REHABILITATION FOR PATIENTS WITH CHRONIC PULMONARY DISEASE (COPD): AN EVIDENCE BASED ANALYSIS." *Ont Health Technol Assess Ser* 12 (2012): 1-47.

Ko 2011

Methods	RCT
Participants	N = 60, rehabilitation: N = 30, no rehabilitation: N = 30
Interventions	8 weeks of rehabilitation, 2-3 times a week, aerobe walking and cycle training
Outcomes	Adverse events, readmissions, breathlessness (mMRC) HRQoL (SGRQ), walking test (6MWT), C-P exercise test (VO2 max)
Notes	6 months follow-up

<u>Risk of bias table:</u>

Bias	Authors' judgements	Support for judgement
Random sequence generation (selection bias)	Low risk	random number generator
Allocation concealment (selection bias)	Unclear risk	Not stated
Blinding of participants and personnel (performance bias)<br>Hospital admission	High risk	Not blinded
Blinding of participants and personnel (performance bias)<br>HRQoL	High risk	Not blinded
Blinding of participants and personnel (performance bias)<br>Mortality	Unclear risk	Nothing stated
Blinding of participants and personnel (performance bias)<br>Walk test	High risk	Not blinded
Blinding of outcome assessment (detection bias)	Low risk	Blinded
Incomplete outcome data (attrition bias)	Low risk	Not detected
Selective reporting (reporting bias)	High risk	Misleading presentation of data on readmissions
Other bias	Low risk	Not detected

Ko 2017

Methods	Study design: Randomized controlled trial<br>Study grouping: Parallel group
Participants	Baseline Characteristics<br>Intervention<br>• *COPD severity (GOLD/MRC score)*: 46.7 (18.3) FEV1, % of pred.<br>• *Male (%)*: 94.4 %<br>• *Age (range)*: 74.9 (7.9) age, years<br><br>Control<br>• *COPD severity (GOLD/MRC score)*: 44.2 (14.7) FEV1, % of pred.<br>• *Male (%)*: 96.7 %<br>• *Age (range)*: 74.6 (8.6) age, years<br><br>Included criteria: Patients who had been admitted with AECOPD to the Prince of Wales Hospital.<br><br>Excluded criteria: Exclusion criteria were: age 40 years; a diagnosis of asthma; chronic lung disease other than COPD (e.g. pneumoconiosis, pulmonary fibrosis); very severe medical illness that would affect the patient's ability to participate in this study (e.g. terminal malignancy); and unable to give informed consent.<br><br>Pretreatment: There was no difference in the demographic characteristics between the groups.
Interventions	Intervention Characteristics<br>Intervention<br>• *Description*: Education and an individualized physical training program to perform at home or a short course of outpatient pulmonary rehabilitation. Phone call from nurse<br>• *Duration (weeks)*: 12 months<br>• *Longest follow up (after end of treatment)*: 12 months after end of treatment<br><br>Control

	• *Description*: Usual care<br>• *Duration (weeks)*: 12 months<br>• *Longest follow up (after end of treatment)*: 12 months after end of treatment
Outcomes	*Mortality, n*<br>• Outcome type: Dichotomous outcome<br>*Quality of life, SD*<br>• Outcome type: Continuous outcome<br>*Readmission due to excerbation, n*<br>• Outcome type: Dichotomous outcome<br>*Walk test, CI*<br>• Outcome type: Continuous outcome<br>*Hospitalization, SD, end of treatment*<br>• Outcome type: Continuous outcome<br>• Data value: Endpoint
Notes	Country: Kina<br>Comments: Trial registration: NCT 01108835<br>Authors name: Fanny W S KO<br>Institution: Devision of Respiratory Medicine, Department of Medicine and Therapeutics, The Chinese University of Hong Kong<br>Email: dschui@cuhk.edu.hk<br>Address: Dept. of Medicine and therapeutics, The Chinese University of Hong Kong. Prince of Wales Hospital, 30-32 Ngan Shing Street, Shatin, New territories. Hong Kong<br>Outcomes<br>Walk test: 6-min, change, longest follow-up. Quality of life: SGRQ, change, longest follow-up. Readmission: adjusted relative risk of readmission for COPD 95% CI, end of treatment. Hospitilazation: days, end of treatment. Death: end of treatment

Risk of bias table:

Bias	Authors' judgements	Support for judgement

Random sequence generation (selection bias)	Low risk	A random number generator was used to assign patients in the intervention or control group
Allocation concealment (selection bias)	Low risk	A computer program (allocation by minimization) was used to assist the randomization of subjects in equal opportunity in either group
Blinding of participants and personnel (performance bias) Hospital admission	Unclear risk	An open study for the patients and therapist, but the research assistant performing lung function, walking tests and questionnaire tests was neither involved in the delivery of patients care nore aware of the randomization
Blinding of participants and personnel (performance bias) HRQoL	Unclear risk	Nothing mentioned
Blinding of participants and personnel (performance bias) Mortality	Unclear risk	Nothing mentioned
Blinding of participants and personnel (performance bias) Walk test	Unclear risk	Nothing mentioned
Blinding of outcome assessment (detection bias)	Unclear risk	Nothing mentioned
Incomplete outcome data (attrition bias)	Low risk	No other apparent sources of bias
Selective reporting (reporting bias)	Low risk	Matches the study protocol
Other bias	Low risk	No other apparent sources of bias

Man 2004

Methods	RCT
Participants	N=42, Rehabilitation: N = 21, Control: N = 21
Interventions	Rehabilitation: Multidisciplinary outpatient pulmonary rehabilitation (within 10 days of discharge) with endurance and strength exercise and patient education for 12 weeks (2 sessions/week). Completion rate of pulmonary rehabilitation of

	85.7% (18 out of 21patients)<br>Usual care: Standard community care with respirologist. Follow-up: 12 weeks
Outcomes	Walking test (SWT), HRQoL, readmissions, days in hospital, mortality
Notes	Follow-up: 12 weeks

Risk of bias table:

Bias	Authors' judgement	Support for judgement
Random sequence generation (selection bias)	Low risk	See Giacomini, M., D. DeJean, and D. Simeonov. "PULMONARY REHABILITATION FOR PATIENTS WITH CHRONIC PULMONARY DISEASE (COPD): AN EVIDENCE BASED ANALYSIS." *Ont Health Technol Assess Ser* 12 (2012): 1-47.
Allocation concealment (selection bias)	Unclear risk	See Giacomini, M., D. DeJean, and D. Simeonov. "PULMONARY REHABILITATION FOR PATIENTS WITH CHRONIC PULMONARY DISEASE (COPD): AN EVIDENCE BASED ANALYSIS." *Ont Health Technol Assess Ser* 12 (2012): 1-47.
Blinding of participants and personnel (performance bias) Hospital admission	High risk	See Giacomini, M., D. DeJean, and D. Simeonov. "PULMONARY REHABILITATION FOR PATIENTS WITH CHRONIC PULMONARY DISEASE (COPD): AN EVIDENCE BASED ANALYSIS." *Ont Health Technol Assess Ser* 12 (2012): 1-47.
Blinding of participants and personnel (performance bias) HRQoL	High risk	See Giacomini, M., D. DeJean, and D. Simeonov. "PULMONARY REHABILITATION FOR PATIENTS WITH CHRONIC PULMONARY DISEASE (COPD): AN EVIDENCE BASED ANALYSIS." *Ont Health Technol Assess Ser* 12 (2012): 1-47.
Blinding of participants and personnel (performance bias) Mortality	Low risk	See Giacomini, M., D. DeJean, and D. Simeonov. "PULMONARY REHABILITATION FOR PATIENTS WITH CHRONIC PULMONARY DISEASE (COPD): AN EVIDENCE BASED ANALYSIS." *Ont Health Technol Assess Ser* 12 (2012): 1-47.
Blinding of participants and personnel (performance bias) Walk test	High risk	See Giacomini, M., D. DeJean, and D. Simeonov. "PULMONARY REHABILITATION FOR PATIENTS WITH CHRONIC PULMONARY DISEASE (COPD): AN EVIDENCE BASED ANALYSIS." *Ont Health Technol Assess Ser* 12 (2012): 1-47.
Blinding of outcome assessment (detection bias)	High risk	See Giacomini, M., D. DeJean, and D. Simeonov. "PULMONARY REHABILITATION FOR PATIENTS WITH CHRONIC PULMONARY DISEASE (COPD): AN EVIDENCE BASED ANALYSIS." *Ont Health Technol Assess Ser* 12 (2012): 1-47.
Incomplete outcome data (attrition bias)	Low risk	See Giacomini, M., D. DeJean, and D. Simeonov. "PULMONARY REHABILITATION FOR PATIENTS WITH CHRONIC PULMONARY DISEASE (COPD): AN EVIDENCE BASED

		ANALYSIS." *Ont Health Technol Assess Ser* 12 (2012): 1-47.
Selective reporting (reporting bias)	Low risk	See Giacomini, M., D. DeJean, and D. Simeonov. "PULMONARY REHABILITATION FOR PATIENTS WITH CHRONIC PULMONARY DISEASE (COPD): AN EVIDENCE BASED ANALYSIS." *Ont Health Technol Assess Ser* 12 (2012): 1-47.
Other bias	Low risk	See Giacomini, M., D. DeJean, and D. Simeonov. "PULMONARY REHABILITATION FOR PATIENTS WITH CHRONIC PULMONARY DISEASE (COPD): AN EVIDENCE BASED ANALYSIS." *Ont Health Technol Assess Ser* 12 (2012): 1-47.

Murphy 2005

Methods	RCT
Participants	N=31, Rehabilitation: N = 16, Control: N = 15
Interventions	Rehabilitation: Supervised home-based pulmonary rehabilitation with endurance and strength exercise for 6 weeks (2 supervised sessions/week and daily unsupervised sessions). Completion rate of pulmonary rehabilitation of 76.9% (10 out of 13 patients)<br>Usual care: Standard community care with respirologist. Follow-up: 26 weeks
Outcomes	Walking test (SWT), breathlessmess, HRQoL, readmissions, exacerbations
Notes	Follow-up: 3 months

<u>Risk of bias table:</u>

Bias	Authors' judgement	Support for judgement
Random sequence generation (selection bias)	Unclear risk	See Giacomini, M., D. DeJean, and D. Simeonov. "PULMONARY REHABILITATION FOR PATIENTS WITH CHRONIC PULMONARY DISEASE (COPD): AN EVIDENCE BASED ANALYSIS." *Ont Health Technol Assess Ser* 12 (2012): 1-47.
Allocation concealment (selection bias)	Low risk	See Giacomini, M., D. DeJean, and D. Simeonov. "PULMONARY REHABILITATION FOR PATIENTS WITH CHRONIC PULMONARY DISEASE (COPD): AN EVIDENCE BASED ANALYSIS." *Ont Health Technol Assess Ser* 12 (2012): 1-47.
Blinding of participants and personnel (performance bias)	Unclear risk	See Giacomini, M., D. DeJean, and D. Simeonov. "PULMONARY REHABILITATION FOR PATIENTS WITH CHRONIC PULMONARY DISEASE (COPD): AN EVIDENCE BASED

Hospital admission		ANALYSIS." *Ont Health Technol Assess Ser* 12 (2012): 1-47.
Blinding of participants and personnel (performance bias) HRQoL	High risk	See Giacomini, M., D. DeJean, and D. Simeonov. "PULMONARY REHABILITATION FOR PATIENTS WITH CHRONIC PULMONARY DISEASE (COPD): AN EVIDENCE BASED ANALYSIS." *Ont Health Technol Assess Ser* 12 (2012): 1-47.
Blinding of participants and personnel (performance bias) Mortality	Low risk	See Giacomini, M., D. DeJean, and D. Simeonov. "PULMONARY REHABILITATION FOR PATIENTS WITH CHRONIC PULMONARY DISEASE (COPD): AN EVIDENCE BASED ANALYSIS." *Ont Health Technol Assess Ser* 12 (2012): 1-47.
Blinding of participants and personnel (performance bias) Walk test	Unclear risk	See Giacomini, M., D. DeJean, and D. Simeonov. "PULMONARY REHABILITATION FOR PATIENTS WITH CHRONIC PULMONARY DISEASE (COPD): AN EVIDENCE BASED ANALYSIS." *Ont Health Technol Assess Ser* 12 (2012): 1-47.
Blinding of outcome assessment (detection bias)	Low risk	See Giacomini, M., D. DeJean, and D. Simeonov. "PULMONARY REHABILITATION FOR PATIENTS WITH CHRONIC PULMONARY DISEASE (COPD): AN EVIDENCE BASED ANALYSIS." *Ont Health Technol Assess Ser* 12 (2012): 1-47.
Incomplete outcome data (attrition bias)	Low risk	See Giacomini, M., D. DeJean, and D. Simeonov. "PULMONARY REHABILITATION FOR PATIENTS WITH CHRONIC PULMONARY DISEASE (COPD): AN EVIDENCE BASED ANALYSIS." *Ont Health Technol Assess Ser* 12 (2012): 1-47.
Selective reporting (reporting bias)	Low risk	See Giacomini, M., D. DeJean, and D. Simeonov. "PULMONARY REHABILITATION FOR PATIENTS WITH CHRONIC PULMONARY DISEASE (COPD): AN EVIDENCE BASED ANALYSIS." *Ont Health Technol Assess Ser* 12 (2012): 1-47.
Other bias	Unclear risk	See Giacomini, M., D. DeJean, and D. Simeonov. "PULMONARY REHABILITATION FOR PATIENTS WITH CHRONIC PULMONARY DISEASE (COPD): AN EVIDENCE BASED ANALYSIS." *Ont Health Technol Assess Ser* 12 (2012): 1-47.

Puhan 2012

Methods	RCT
Participants	N=36, early rehabilitation: N = 19, late rehabilitation: N = 17

Interventions	12-week program, in or outpatient rehabilitation center, 24 sessions (range 18-36), including both endurance and strength, and education
Outcomes	Exacerbation rate over 18 months, HRQoL (CRQ), breathlessness (mMRC)
Notes	

Risk of bias table:

Bias	Authors' judgement	Support for judgement
Random sequence generation (selection bias)	Low risk	See Puhan, Milo A., et al. "Pulmonary rehabilitation following exacerbations of chronic obstructive pulmonary disease." *Cochrane Database Syst Rev* 10.10 (2011).
Allocation concealment (selection bias)	Low risk	See Puhan, Milo A., et al. "Pulmonary rehabilitation following exacerbations of chronic obstructive pulmonary disease." *Cochrane Database Syst Rev* 10.10 (2011).
Blinding of participants and personnel (performance bias) Hospital admission	High risk	See Puhan, Milo A., et al. "Pulmonary rehabilitation following exacerbations of chronic obstructive pulmonary disease." *Cochrane Database Syst Rev* 10.10 (2011).
Blinding of participants and personnel (performance bias) HRQoL	High risk	See Puhan, Milo A., et al. "Pulmonary rehabilitation following exacerbations of chronic obstructive pulmonary disease." *Cochrane Database Syst Rev* 10.10 (2011).
Blinding of participants and personnel (performance bias) Mortality	Low risk	See Puhan, Milo A., et al. "Pulmonary rehabilitation following exacerbations of chronic obstructive pulmonary disease." *Cochrane Database Syst Rev* 10.10 (2011).
Blinding of participants and personnel (performance bias) Walk test	High risk	See Puhan, Milo A., et al. "Pulmonary rehabilitation following exacerbations of chronic obstructive pulmonary disease." *Cochrane Database Syst Rev* 10.10 (2011).
Blinding of outcome assessment (detection bias)	Unclear risk	See Puhan, Milo A., et al. "Pulmonary rehabilitation following exacerbations of chronic obstructive pulmonary disease." *Cochrane Database Syst Rev* 10.10 (2011).
Incomplete outcome data (attrition bias)	Low risk	See Puhan, Milo A., et al. "Pulmonary rehabilitation following exacerbations of chronic obstructive pulmonary disease." *Cochrane Database Syst Rev* 10.10 (2011).
Selective reporting (reporting	Low risk	See Puhan, Milo A., et al. "Pulmonary rehabilitation following exacerbations of chronic

bias)		obstructive pulmonary disease." *Cochrane Database Syst Rev* 10.10 (2011).
Other bias	Low risk	See Puhan, Milo A., et al. "Pulmonary rehabilitation following exacerbations of chronic obstructive pulmonary disease." *Cochrane Database Syst Rev* 10.10 (2011).

Revitt 2018

Methods	Study design: Randomized controlled trial<br>Study grouping: Parallel group
Participants	Baseline Characteristics<br>Intervention<br>• *COPD severity (GOLD/MRC score)*: 51.04 (20.46), FEV1 % of predicted.<br>• *Male (%)*:<br>• *Age (range)*: 64.32 (7.37)<br>Control<br>• *COPD severity (GOLD/MRC score)*: 52.33 (17.53), FEV1 % of predicted<br>• *Male (%)*:<br>• *Age (range)*: 65.8 (7.24)<br><br>Included criteria: Inclusion criteria were confirmed diagnosis of COPD prior to current admission and an increase in self-reported breathlessness on exertion.<br><br>Excluded criteria: Exclusion criteria were inability to provide informed consent; acute cardiac event; and the presence of musculoskeletal, neurological and psychiatric co-morbidities that would prevent the delivery of PR.<br><br>Pretreatment: Both groups were well matched for age, Lung function and exercise capacity. Randomization was not equal across both arms with N = 24 in the early PR group and N = 12 in the D-PEPR group.
Interventions	Intervention Characteristics<br>Intervention<br>• *Description*: Occurred within 4 weeks of discharge. PR was delivered twice weekly for 6 weeks, with each session being 2 hours. It consisted of individualized aerobic and resistance exercises and education which covered topics including chest

	clearance and energy conservation.<br>• *Duration (weeks)*: 6 weeks<br>• *Longest follow up (after end of treatment)*: End of treatment<br><br>Control<br>• *Description*: 7 weeks after a control period. PR was delivered twice weekly for 6 weeks, with each session being 2 hours. It consisted of individualized aerobic and resistance exercises and education which covered topics including chest clearance and energy conservation.<br>• *Duration (weeks)*: 6 weeks<br>• *Longest follow up (after end of treatment)*: End of treatment
Outcomes	*Dropouts, n*<br>• Outcome type: DichotomousOutcome<br><br>*Shuttle Walk test, end of treatment*<br>• Outcome type: ContinuousOutcome
Notes	

Risk of bias table:

Bias	Authors' judgement	Support for judgement
Random sequence generation (selection bias)	Unclear risk	Nothing mentioned
Allocation concealment (selection bias)	Low risk	Judgement Comment: Sealed envelope technique
Blinding of participants and personnel (performance bias) Hospital admission	Unclear risk	Nothing mentioned
Blinding of participants and personnel (performance bias) HRQoL	Unclear risk	Nothing mentioned

Blinding of participants and personnel (performance bias) Mortality	Unclear risk	Nothing mentioned
Blinding of participants and personnel (performance bias) Walk test	Unclear risk	Nothing mentioned
Blinding of outcome assessment (detection bias)	Unclear risk	Judgement Comment: Nothing mentioned
Incomplete outcome data (attrition bias)	Low risk	Judgement Comment: Dropouts have been accounted for
Selective reporting (reporting bias)	High risk	Quote: "Health-related quality of life measures were gathered, but on analysis, there were insufficient complete data sets to enable accurate analysis so this has not been reported."
Other bias	High risk	Quote: "As a result of the original sample number not being met in the allocated time and lower than anticipated uptake and retention issues, the trial was terminated prematurely and was deemed a failed trial."

Seymour 2010

Methods	RCT
Participants	N = 60, Rehabilitation: N = 30, Control: N = 30
Interventions	Rehabilitation: Within a week after hospital discharged, outpatient pulmonary rehabilitation twice-weekly exercise (limb strengthening and aerobic activities) and education sessions, for 8 weeks. Completion rate of pulmonary rehabilitation of 77% (23 out of 30). Patients were provided with general information about COPD and offered outpatient appointments with general practitioner or respiratory team. Follow-up: 12 weeks<br><br>Usual care: Patients were provided with general information about COPD and offered outpatient appointments with general practitioner or respiratory team. Not referred further. Follow-up: 12 weeks
Outcomes	Readmissions due to exacerbations, muscle strength, walking test (SWT), HRQoL
Notes	Follow-up: 3 months after admission

Risk of bias table:

Bias	Authors' judgement	Support for judgement
Random sequence generation (selection bias)	Unclear risk	See Giacomini, M., D. DeJean, and D. Simeonov. "PULMONARY REHABILITATION FOR PATIENTS WITH CHRONIC PULMONARY DISEASE (COPD): AN EVIDENCE BASED ANALYSIS." *Ont Health Technol Assess Ser* 12 (2012): 1-47.
Allocation concealment (selection bias)	Unclear risk	See Giacomini, M., D. DeJean, and D. Simeonov. "PULMONARY REHABILITATION FOR PATIENTS WITH CHRONIC PULMONARY DISEASE (COPD): AN EVIDENCE BASED ANALYSIS." *Ont Health Technol Assess Ser* 12 (2012): 1-47.
Blinding of participants and personnel (performance bias) Hospital admission	High risk	See Giacomini, M., D. DeJean, and D. Simeonov. "PULMONARY REHABILITATION FOR PATIENTS WITH CHRONIC PULMONARY DISEASE (COPD): AN EVIDENCE BASED ANALYSIS." *Ont Health Technol Assess Ser* 12 (2012): 1-47.
Blinding of participants and personnel (performance bias) HRQoL	High risk	See Giacomini, M., D. DeJean, and D. Simeonov. "PULMONARY REHABILITATION FOR PATIENTS WITH CHRONIC PULMONARY DISEASE (COPD): AN EVIDENCE BASED ANALYSIS." *Ont Health Technol Assess Ser* 12 (2012): 1-47.
Blinding of participants and personnel (performance bias) Mortality	Low risk	See Giacomini, M., D. DeJean, and D. Simeonov. "PULMONARY REHABILITATION FOR PATIENTS WITH CHRONIC PULMONARY DISEASE (COPD): AN EVIDENCE BASED ANALYSIS." *Ont Health Technol Assess Ser* 12 (2012): 1-47.
Blinding of participants and personnel (performance bias) Walk test	High risk	See Giacomini, M., D. DeJean, and D. Simeonov. "PULMONARY REHABILITATION FOR PATIENTS WITH CHRONIC PULMONARY DISEASE (COPD): AN EVIDENCE BASED ANALYSIS." *Ont Health Technol Assess Ser* 12 (2012): 1-47.
Blinding of outcome assessment (detection bias)	Unclear risk	See Giacomini, M., D. DeJean, and D. Simeonov. "PULMONARY REHABILITATION FOR PATIENTS WITH CHRONIC PULMONARY DISEASE (COPD): AN EVIDENCE BASED ANALYSIS." *Ont Health Technol Assess Ser* 12 (2012): 1-47.
Incomplete outcome data (attrition bias)	Low risk	See Giacomini, M., D. DeJean, and D. Simeonov. "PULMONARY REHABILITATION FOR PATIENTS WITH CHRONIC PULMONARY DISEASE (COPD): AN EVIDENCE BASED ANALYSIS." *Ont Health Technol Assess Ser* 12 (2012): 1-47.
Selective reporting (reporting bias)	Low risk	See Giacomini, M., D. DeJean, and D. Simeonov. "PULMONARY REHABILITATION FOR PATIENTS WITH CHRONIC PULMONARY DISEASE (COPD): AN EVIDENCE BASED ANALYSIS." *Ont Health Technol Assess Ser* 12 (2012): 1-47.
Other bias	Unclear risk	See Giacomini, M., D. DeJean, and D. Simeonov. "PULMONARY REHABILITATION FOR

		PATIENTS WITH CHRONIC PULMONARY DISEASE (COPD): AN EVIDENCE BASED ANALYSIS." *Ont Health Technol Assess Ser* 12 (2012): 1-47.

Troosters 2000

Methods	RCT
Participants	43 COPD patients (mean age 62 years, 85%males, FEV1=39%predicted) after inpatient treatment for acute exacerbation
Interventions	Rehabilitation: Outpatient pulmonary rehabilitation with endurance and strength exercise for 6 months (3 sessions/week in first 3 months, then 2/week). Completion rate of pulmonary rehabilitation of 70.8% (17 out of 24 patients)<br>Usual care: Standard community care with respirologist (not further specified). Follow-up: 208 weeks
Outcomes	6MWD, mortality
Notes	

<u>Risk of bias table:</u>

Bias	Authors' judgement	Support for judgement
Random sequence generation (selection bias)	Unclear risk	See Puhan, Milo A., et al. "Pulmonary rehabilitation following exacerbations of chronic obstructive pulmonary disease." *Cochrane Database Syst Rev* 10.10 (2011).
Allocation concealment (selection bias)	Unclear risk	See Puhan, Milo A., et al. "Pulmonary rehabilitation following exacerbations of chronic obstructive pulmonary disease." *Cochrane Database Syst Rev* 10.10 (2011).
Blinding of participants and personnel (performance bias) Hospital admission	Unclear risk	See Puhan, Milo A., et al. "Pulmonary rehabilitation following exacerbations of chronic obstructive pulmonary disease." *Cochrane Database Syst Rev* 10.10 (2011).
Blinding of participants and personnel (performance bias) HRQoL	High risk	See Puhan, Milo A., et al. "Pulmonary rehabilitation following exacerbations of chronic obstructive pulmonary disease." *Cochrane Database Syst Rev* 10.10 (2011).
Blinding of participants and personnel (performance bias) Mortality	Low risk	See Puhan, Milo A., et al. "Pulmonary rehabilitation following exacerbations of chronic obstructive pulmonary disease." *Cochrane Database Syst Rev* 10.10 (2011).
Blinding of participants and	Unclear risk	See Puhan, Milo A., et al. "Pulmonary rehabilitation following exacerbations of chronic

personnel (performance bias) Walk test		obstructive pulmonary disease." *Cochrane Database Syst Rev* 10.10 (2011).
Blinding of outcome assessment (detection bias)	Unclear risk	See Puhan, Milo A., et al. "Pulmonary rehabilitation following exacerbations of chronic obstructive pulmonary disease." *Cochrane Database Syst Rev* 10.10 (2011).
Incomplete outcome data (attrition bias)	Low risk	See Puhan, Milo A., et al. "Pulmonary rehabilitation following exacerbations of chronic obstructive pulmonary disease." *Cochrane Database Syst Rev* 10.10 (2011).
Selective reporting (reporting bias)	Low risk	See Puhan, Milo A., et al. "Pulmonary rehabilitation following exacerbations of chronic obstructive pulmonary disease." *Cochrane Database Syst Rev* 10.10 (2011).
Other bias	Low risk	See Puhan, Milo A., et al. "Pulmonary rehabilitation following exacerbations of chronic obstructive pulmonary disease." *Cochrane Database Syst Rev* 10.10 (2011).
