# Supplementary material for: Lower mortality after early supervised pulmonary rehabilitation following COPD-exacerbations: a systematic review and meta-analysis
Source: BMC Pulm Med. 2018 Sep 15;18:154. doi: 10.1186/s12890-018-0718-1 (PMC6139159; doi:10.1186/s12890-018-0718-1)
Supplement: Supplementary file 5 — Table S1. Extensiveness of the PR programs in the included studies. (PDF 23 kb) [file 12890_2018_718_MOESM5_ESM.pdf]

Additional file 5.

We followed the statements and guidelines from BTS [26], ERS/ATS [10], and as described in Puhan *et al.* [14]. We did not upgrade or downgrade the extent of the PR program if the program was in line with the statements and guidelines. However, we upgraded or downgraded, respectively, the extent of the programs if some components were less or exceeded what these guidelines recommends. We considered PR programs to be extensive if: participant followed, on average, at least 16 exercise sessions, calculated as the total number of possible exercise session times the attendance rate, as recommended by ERS/ATS; the PR program included 2 to 3 exercise sessions per week, as recommended by BTS and ERS/ATS; exercise training included at least endurance exercise (± strength exercise), as recommended by BTS and ERS/ATS; or exercise sessions were supervised, as recommended by BTS and ERS/ATS.

Similar to Puhan *et al.* [14], we used the GRADE approach and downgraded the extent of PR program for the following reasons: if the total number of training sessions was between 10 and 15 (by -1) and less than 10 sessions (by -2); fewer than 2 sessions per weeks (by -1); if exercise training only included strength training or less than 20 min of endurance training per sessions etc. (by -1). The extent of PR was upgraded if: the number of sessions was greater than 30 (by +1) or PR included an extensive self-management program (by +1).

Table 1. Extensiveness of PR programs in included studies.

Study	Number of sessions	Sessions per week	PR program	Supervision of training	Extent of PR
Behnke 2000	+1[a]	-	-	-1[b]	Extensive
Daabis 2017	-	-	-	-	Extensive
Deepak 2014	Unclear[c]	Unclear	-	-	Unclear
Eaton 2009	-1[d]	-	+1[e]	-	Extensive
Kirsten 1998	-	-	-	-	Extensive
Ko 2011	-	-	-	-	Extensive
Ko 2017	-	-	+1[e]	-1[b]	Extensive

Man 2004	-1[d]	-	+1[e]	-	Extensive
Murphy 2005	-1[d]	-	-	-	Moderate extensive
Puhan 2012	-	-	+1[e]	-	Extensive
Revitt 2018	-2[f]	-	-	-	Slightly extensive
Seymour 2010	-1[d]	-	+1[e]	-	Extensive
Troosters 2000	+1[a]	-	-	-	Extensive

[a] >30 exercise training sessions.
[b] Some training sessions were unsupervised (home-based training period).
[c] A 12-week exercise intervention, but unclear number of sessions.
[d] 10 to 15 training sessions.
[e] Comprehensive self-management education.
[f] <10 exercise training sessions.
